# A Novel Agro‐Waste Formulated Medium Enhanced the Growth of Electrogenic *Enterobacter* Species Isolated Using Microbial Fuel Cell System: Response Surface Methodology Approach

**DOI:** 10.1111/1758-2229.70232

**Published:** 2025-11-07

**Authors:** Getachew Bantihun, Andualem Mekonnen, Venkata Kotakadi, Seid Mohammed

**Affiliations:** ^1^ Department of Applied Biology Adama Science and Technology University Adama Ethiopia; ^2^ Center for Environmental Science Addis Ababa University Addis Ababa Ethiopia; ^3^ DST‐PURSE Centre Sri Venkateswara University Tirupati Andhra Pradesh India

**Keywords:** biofilm, cell dry weight, electrogenic bacteria, microbial fuel cell, response surface methodology

## Abstract

Our study aims to identify electrogenic bacteria and optimise culture conditions using different commercial and agro‐industrial wastes as a sole carbon source. Potential candidates of electrogenic bacteria isolates (EBIs) were screened from anode‐developed biofilm in a double‐chambered microbial fuel cell (MFC) bioreactor system. Characterisation using cyclic voltammetry (CV) showed that the isolated bacteria had a potential bio‐electrochemical property. Statistical techniques were used, including response surface methodology (RSM) with a central composite design (CCD). The highest cell growth, measured by optical density at 600 nm (OD_600nm_) (1.1407 ± 0.00316) and cell dry weight (CDW) (0.02135 ± 0.00152 g/L), was obtained when commercial carbon glucose was used. Cost‐effective, barley bran formulated media resulted in maximum growth, OD_600nm_ 1.52167 ± 0.03476 and CDW with 0.01541 ± 0.000071 g/L. The RSM optimised condition achieved a 32.3% fold increase of cell growth yield (OD_600nm_) compared to unoptimised conditions. This is the first study to use 16S rRNA gene sequencing from anode biofilm to identify native *Enterobacter* species. In conclusion, the recently discovered isolate exhibited growth conditions between 18°C and 52°C, pH 3 and pH 11, and resistance to high salt concentrations (0.332 M NaCl). It might therefore be considered a potentially versatile biocatalyst candidate for MFC applications.

## Graphical abstract

Electrogenic isolate, *Enterobacter* sp. DSAAI‐4 exhibited broad substrate range utilisation ability and high salt tolerance. Use of agro‐industrial waste barley bran for culture growth was promising and low cost.

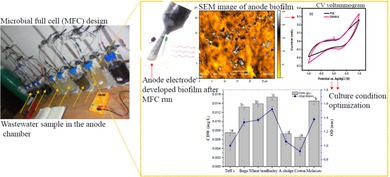

## 1 Introduction

A microbial fuel cell (MFC) is a promising innovative kind of bio‐electrochemical reactor system that uses electrogenic bacteria activity to produce electricity and degrade pollutants. Reports have shown that MFCs are an environmentally benign method of treating a variety of wastewater and that they can also convert energy with the help of biocatalysts (Guo et al. 2020). In a MFC bioreactor system, electrogenic bacteria play a vital role in oxidation activities on the anode (Supporting Information: Fig. A_2_), which is followed by the release of electrons as byproducts as shown in the anodic oxidation reaction Equation (1) below.

(1) $$\begin{array}{c} \text{Biodegradable organic matter}+H_{2}O\\ +\text{Electrogenic bacteria}➔{\mathrm{CO}}_{2}+H^{+}+e^{-} \end{array}$$

As it was described by Pandya et al. (2024), MFCs are bioreactors that harness the catalytic activity of electrogenic bacteria in anaerobic environments to transform the chemical bonding energy of organic compounds into electrical energy. More importantly, MFCs have demonstrated tremendous promise as multifunctional factories for addressing the three main sustainability problems facing the globe today: wastewater treatment, global warming, and energy security (Shajid et al. 2025).

Electrogenic bacteria are a unique group of microorganisms that possess the remarkable ability to transfer electrons extracellularly across the cell envelope to the final electron acceptor found in the anode of the MFC bioreactor (Tahernia et al. 2020). These bacterial cells grow under anaerobic conditions at the anode surface of the MFCs and form a biofilm (Hemdan et al. 2023). Interestingly, through the cellular respiration process, electrogenic bacteria degrade organic substrates on the anode surface to produce electric current from energy‐rich bonds of organic substrates, in addition to degrading pollutants (Aiyer 2020). More importantly, for the reuse of cost‐effective biomass wastes, electroactive bacteria play a great role in converting the chemical energy from complex biomass into electrical energy (Zhou et al. 2023).

Exoelectrogenic bacteria use the process of cellular respiration to break down organic or inorganic substrates at the anode surface. The respiratory response in electrogenic bacteria takes place in the cell membrane, which houses all of the machinery, including proteins and enzymes. This results in the production of electric current, which is then transported via specialised electron transport chains (Aiyer 2020). Secondary metabolites produced by bacteria, including flavones, pyocyanins, and quinones can function as endogenous soluble electron shuttles. These metabolites interact with cytochromes, facilitating the generation of energy (Nawaz et al. 2020). It requires transforming the produced electrons from inside the cells to the anode surface in anoxic conditions to produce electric current (He et al. 2021). During the transfer of electrons to the anode surface, bacteria can also use electron shuttles (Kouzuma et al. 2014) and proteinaceous filaments (Mahmoud et al. 2022). These bacteria include iron‐reducing Geobacter species, *Rhodoferax ferrireducens* , *Pseudomonas aeruginosa* , Shewanella species, *Aeromonas hydrophila* , *Clostridium butyricum* , and *Enterococcus* species (Mahidhara et al. 2021).

Apart from their potential application as biocatalysts in MFCs, cellulose‐producing electrogenic bacteria are reported as a potential precursor for electrode material to protect from corrosion that limits conductive biofilm establishment (Hayta et al. 2021). The sustainable operation and success of bio‐electrochemical systems lie in electroactive biofilm formation by electroactive bacteria. Notably, Schneider et al. (2023) reported that biofilm formation has a significant contribution to adhering to the electrode so that it enables a smooth process in electron transfer by the electrogenic bacteria. The significant contribution of biofilm‐forming EBIs to enhance electron transfer and its capabilities, allowing direct contact with the electrode surface, has been broadly discussed (Conners et al. 2022). In spite of this, biofilm formation is a complex phenomenon; it is evident that integrated aggregates of electrogenic bacterial cells are beneficial for wastewater treatment using cost‐effective anaerobic MFCs (Amankwah et al. 2021).

Furthermore, using inexpensive, cost‐effective agro‐industrial waste as a source of nutrients is particularly important from a variety of perspectives, including economic, sustainable, and environmentally friendly, as it lowers the environmental pollution load. However, to the best of our knowledge there is no study with barley bran (BB) as an alternative cost‐effective agro‐waste carbon source for electrogenic bacteria growth. More importantly, the use of alternative, easily available, cheap substrates can have direct benefits for environmental health issues. Therefore, it is highly imperative to understand which of these commercial and cheap agro‐industrial waste substrates best promotes better growth of EBIs. For efficient performance of MFCs using biocatalysts, optimisation of their growth condition against carbon sources, nitrogen sources, cheap agro‐industrial waste as a substrate source, and other physical conditions is highly needed. Besides the conventional one factor‐at‐a‐time optimisation method, the response surface methodology (RSM) is highly effective in determining optimal growth conditions by providing interactive effects at a single time, which minimises the experimental error of either excess use or limited conditions during growth medium formulations.

Electrochemical performance is mainly dependent on electrogenic bacteria with their promising future solutions in wastewater treatment and energy generation; hence, understanding the growth conditions of electrogenic bacteria with different alternative carbon sources, growth temperatures, pH, salt concentrations, substrate concentration and trace elements is very critical. So far substrate utilisation and physical growth conditions using different substrate types have not been done for the newly identified electrogenic *Enterobacter* sp. DSAAI‐4. There is no study conducted with different cost‐effective and eco‐friendly agro‐industrial waste types to propose as an alternative growth medium formulation for EBIs. Furthermore, RSM approach optimisation studies have not been reported so far for the currently isolated EBIs biocatalyst to be applicable in MFCs. Therefore, the aim of this study was to explore a bacterium with electrogenic features from different industrial and domestic wastewaters and to further study substrate utilisation through optimisation analysis.

This study looked at using different agro‐industrial and sludge waste as a source of carbon to improve the growing conditions of electrogenic bacteria. In vitro growth of the potential electrogenic bacterium isolate was examined on cost‐effective agro‐industrial wastes such as bagasse, BB, brewery activated sludge, cotton waste, molasses, teff straw and wheat bran. In addition, optimisation of growth conditions for a potent isolate was done using the RSM approach for the first time. Furthermore, in this study characterisation of electrogenic features was done using rapid, highly sensitive cyclic voltammetry, and their biofilm‐forming capacities were also investigated. This study presents a new approach for isolating native electrogenic bacteria from anode‐developed biofilms in MFC using real wastewater, addressing a critical lack of research. The newly isolated electrogenic bacteria exhibit adaptable growth on diverse substrates and possess electrochemical properties suitable for wastewater treatment. A novel growing medium derived from agro‐industrial waste offers improved cost‐effectiveness and sustainability. This innovative strategy lowers production costs while addressing environmental concerns by recycling waste materials that could otherwise contribute to pollution. Furthermore, a new approach RSM validates experimental work, providing valuable insights for future research.

## 2 Materials and Methods

### 2.1 Sample Collection

A composite sampling method was employed to collect various industrial and municipal wastewater, activated sludge, and soil samples from multiple locations (Supporting Information: Fig. A_1_) by following the literature (Khumalo et al. 2022). Sample types presented in an additional file (Supporting Information: Table A_1_) were transported to the microbiology laboratory using an icebox (Cosmoplast k/c picnic MFIBXX080CG, UAE) and kept at 4°C for further study.

### 2.2 Microbial Fuel Cell Reactor Set‐Up for the Experiment

A double‐chambered MFC consisting of an anode and cathode was constructed using a 500 mL total capacity Schott Duran glass bottle, and the chambers were connected with the salt bridge agar as previously reported by Roy et al. (2023). All glass bottles were sterilised for 15 min at 121°C (Autoclave, D‐3/058/1146, India). In the cathode chamber, an oxidising agent, KMnO_4_ (25 mM), was added. Pencil rod graphite electrodes (PGE) were inserted through drilled holes of each chamber (Supporting Information: Fig. A_2_).

### 2.3 EBIs Isolation From Anode Electrode

At the end of the experimental process, the anode electrodes were withdrawn from the MFC reactors and then carefully washed with 0.1 M phosphate buffer saline at pH 7.0 to remove any adhered debris. The scraped anodic biofilm was deposited in a sterile 50 mL tube containing 10 mL of 50 mM PBS (Hemdan et al. 2023). The obtained suspension was serially diluted up to 10^−6^, and from each dilution, 0.1 mL was plated on selective minimal chromogenic medium (MCM) (Supporting Information: Table A_2_) following the methods (Nazeer and Fernando 2022). All petri plates were incubated at 35°C for 24 h under anaerobic conditions using an anaerobic jar.

### 2.4 Biochemical Characterisation Against EBIs

All experimental works were performed following the standard methods of the Society of American Bacteriologists Manual of Microbiological Methods, Bergey's Manual of Determinative Bacteriology, and the respective media manufacturer's directions. After growth, colony morphology, including form, elevation, margin, and pigmentation was examined. Gram characteristics were conducted on 24‐h‐old cultures according to the standard staining technique as described by the American Society for Microbiology (Smith and Hussey 2016).

Biochemical tests such as the catalase test (Iwase et al. 2013), citrate utilisation (Hadi and Dewi 2019), cytochrome oxidase (Shields and Cathcart 2013), indole production test using multi SIM (Bhutia et al. 2021), methyl‐red (MR) and Voges‐Proskauer (VP) (Bhutia et al. 2021), triple sugar iron (Lehman 2005), and urease hydrolysis test (Brink 2010) were carried out against EBIs.

### 2.5 Extracellular Enzymatic Assays

The pure EBIs were tested for proteolytic, amylolytic, and cellulolytic enzymatic activity. Accordingly, proteolytic activity was studied using nutrient agar (NA) (Supporting Information: Table A_2_) enriched with 1% skim milk (w/v) and bromocresol green dye (BCG, 0.0015%, w/v) (Vijayaraghavan et al. 2013). Similarly, carboxymethylcellulose (CMC, 1%, w/v) (Supporting Information: Table A_2_) was supplemented in NA for screening of cellulase‐producing EBIs (Chantarasiri 2015). The extracellular amylase enzyme production profile for EBIs was also studied. Accordingly, NA medium was enriched with 1% (w/v) starch, pure isolates were inoculated, and plates were incubated at 35°C for 48 h.

### 2.6 Characterisation of MFCs Anode Biofilm Using Scanning Electron Microscope (SEM)

MFCs anode biofilm characterisation was performed according to the methods of (Ishii et al. 2012; Thulasinathan et al. 2021) using SEM to confirm the microbial biofilm formation at the electrode's surface. Briefly, to observe the surface morphology of the electrode biofilm and colonisation of cells, the biofilm‐grown electrodes were carefully removed. Then, small sections of the anode electrode were cut off from the anode chamber using a sterile scalpel and rinsed with sterile distilled water and further washed with sterile 0.1 M phosphate buffer saline (pH 7.0) to remove the excess matter. Then, the samples were fixed for 2 h with a 2.5% (v/v) glutaraldehyde solution following the previous methods (Kumar et al. 2015).

### 2.7 In Vitro Phenotypic Biofilm Detection

#### 2.7.1 Congo Red Agar Biofilm Assay

Investigation of biofilm formation was carried out (Hashem et al. 2017). The Congo red agar method (CRAM) was carried out using brain heart infusion agar (BHIA) (Supporting Information: Table A_2_) supplemented with 5% (w/v) sucrose, and pH was maintained at 7.0. Plates were streaked using sterile loops and incubated for 48 h at 37°C following the previous methods (Harika et al. 2020).

#### 2.7.2 Microtiter‐Plate Test for Biofilm Assay

Bacterial isolates were grown overnight in 10 mL sterile trypticase soy broth (TSB) containing tubes at 30°C for 24 h. Then, freshly grown (24 h) 60 μL of cell suspension was inoculated in 140 μL of TSB medium in each well, and 200 μL of autoclaved distilled water was added in peripheral wells to reduce the water loss consequent to an edge effect (Mansoury et al. 2021). Then, microtiter plates were covered and incubated for 48 h at 35°C. Indeed, the plates were stained with 0.2 mL of 0.1% (w/v) crystal violet and left for 10 min at room temperature. Biofilms were dissolved with 200 μL of 33% (v/v) glacial acetic acid following previously described methods by Dwivedi and Singh (2016) and the optical density (OD) of the biofilm was quantified using a micro plate reader (OR‐9710220, DAS, Italy) at a wavelength of 570 nm. The experiment was performed in triplicate. Each isolate was classified as follows: weak biofilm producer OD ≤ 2 × ODc, moderate biofilm producer OD ≤ 4 × ODc and strong biofilm producer OD > 4 × ODc (Stepanović et al. 2004). Where ODc is the OD cut‐off value, which is equal to the summation of the average value of the negative control of OD and three times the standard deviation of the negative control (Stepanović et al. 2004).

#### 2.7.3 Tube Method Biofilm Assay

In this method, 2 mL of sterile Trypticase soy broth (TSB) was inoculated with a loopful of electrogenic bacterial isolates and incubated for 48 h at 35°C. Tubes were then stained with (0.1%, w/v) crystal violet for 10 min and washed with sterile deionised water. For quantification, 2 mL of 33% (v/v) glacial acetic acid was added to dissolve biofilm and quantified using a UV–visible spectrophotometer (OPTIZEN 2120UV, Moscow) at 570 nm, and phenotypic detection with ring formation after washing and staining was a positive result following previously reported methods (Basnet et al. 2023).

### 2.8 Experimental Design for Screening and Optimisation for Efficient Growth Conditions

#### 2.8.1 Culture Cultivation Condition on Commercial Carbon and Nitrogen Sources

The effect of growth conditions for the potential electrogenic isolate was examined in vitro using different carbon and nitrogen sources. More briefly, the effect of fructose, glucose, mannitol and sucrose on growth condition was studied using OD (OD_600nm_) and cell biomass (CDW, g/L). In brief, following the previous method by Almihyawi et al. (2024) each carbon source (1% w/v) was supplemented separately to Erlenmeyer flasks containing 100 mL of minimal salt medium (MSM). The cell growth was measured using a UV–visible spectrophotometer (OPTIZEN 2120UV, Moscow) at OD_600nm_. Similarly, the culture cells were harvested after growth by centrifugation at 10, 000 rpm at room temperature for 10 min using a centrifuge (Eltek, Maalab Scientific Equipment an ISO 9001, India). The supernatant was discarded, and the cell pellets were dried in the oven (DRD360DB, China) at a temperature of 50°C for 30 min and the mass was determined using an electronic balance (D450028152 Shimadzu, Japan). The effect of different nitrogen sources on the growth of EBIs was also studied. Accordingly, following the methods described earlier, 0.6% (w/v) each of ammonium sulfate, peptone, potassium nitrate, urea and yeast extract were formulated with MSM medium with the same protocol as described in the case of carbon sources (Gupta and Khare 2007).

#### 2.8.2 Culture Cultivation on Alternative Cost Effective Agro‐Industrial By‐Products

To employ these alternative carbon sources, pretreatment was carried out; accordingly except for molasses and activated sludge, all were meticulously washed with distilled water, dried at room temperature and ground with an electric blender (Shanghai Jinkle, Scientific Instrument, China) into fine powder followed by sieving with a mesh size of 1.0 mm. Furthermore, each ground agro‐industrial waste residue (100 g, w/v) was individually soaked in distilled water until complete boiling was reached. Briefly, thermal pretreatment of molasses was done by incubating 100 mL of molasses in a 90°C water bath for 1 h (Mawarda et al. 2018). Finally, 1% (w/v) of molasses supernatant was supplemented as a carbon source to the MSM medium. Activated overnight cell culture (100 μL) was inoculated into MSM medium supplemented with formulated carbon sources with a working volume of 100 mL and incubated for 48 h in a shaker incubator at 130 rpm. The growth effects among various treated carbon sources were evaluated and their corresponding OD_600nm_ and CDW (g/L) were recorded.

#### 2.8.3 Optimisation of the Growth Condition Using Response Surface Methodology (RSM) Approach

The growth conditions of EBIs with the interaction of various physical and chemical parameters (pH, temperature and salt concentration) were performed using central composite design (CCD) of RSM. Briefly, optimisation of fermentation conditions for isolate DSAAI‐4 was studied using Design Expert 13 (Stat‐Ease Inc., Minneapolis, MN, USA). In this approach, a three‐level three‐factor CCD‐based RSM was designed using one variable at a time, while other variables were kept constant and the significant effect of interactions on cultivation conditions was examined following the previous method with modifications (Melini et al. 2023). With this statistical approach, the influence of independent variables on growth conditions in terms of OD_600nm_ and CDW (g/L) was set as a response and analyzed to propose the best optimum growth condition. The experiment comprised 20 runs, of which 6 were central, 6 axial and 8 factorial runs (Table 1). To understand the significance of all factors, statistical computation of the *F* value at a probability (*p*) of 0.05 was evaluated following the previous methods (Melini et al. 2023).

**TABLE 1:** Factors and their levels used in the central composite design.

Factors	Levels			Central composite design (CCD) parameters	
	Low	Central	High		
Temperature (°C)	18	35	52	Axial type	6
pH	0.3	7	13	Center point	6
Salt concentration (g/L)	0.2	8.5	19.4	Factorial	8
				Total runs	20

#### 2.8.4 Verification of the Model With the Actual Experimental Work

To further validate experimentally, broth culture (100 mL) was cultivated in flask fermentation (250 mL capacity) with the selected theoretical set of conditions and bacterial growth was monitored through OD_600nm_ and recorded as an actual response. Individually prepared and inoculated media were incubated for 48 h with a specific predicted incubation temperature (33°C). With this, theoretically predicted values and actual experimental responses were determined using the lack of fit test (F test). Similarly, optimum conditions were experimentally performed, and the value of CDW (g/L) was compared with that of the theoretical results. The quality of fit statistics were evaluated using both predicted and adjusted correlation coefficients (*R* ^2^), where the model was accepted with less than 20% difference of adequate precision among them (Rocha et al. 2023).

### 2.9 Identification of Potential Electrogenic Isolates

#### 2.9.1 Rapid Identification of EBIs Based on Protein Profile

Pure EBIs were rapidly identified from the anode‐developed biofilm region using a biotyper MALDI‐TOF MS system coupled with a mass spectrometer (MS) based on the manufacturers' instructions (Biotyper operating system, Zybio‐EXS3000, China) at Wudassie Diagnostic Center. Identification score followed the manufacturer interpretation criteria: a logarithmic score value (LSV) of > 2.000 indicated species‐level identification, LSV of 1.700 to 1.999 indicated identification to the genus level, and LSV of < 1.700 was interpreted as no identification (Table 4).

#### 2.9.2 16S rRNA Gene Analysis for EBIs

In this study a 16S rRNA sequence against a pure isolate of DSAAI‐4 was performed based on its potential performance as evidenced by CV and polarisation studies. Accordingly, the DNA for the selected isolate was extracted using the Biobee Spin EXpure Microbial DNA isolation kit developed by Bogar Bio Bee Stores Pvt. Ltd. The quality of the DNA extract was checked by using 1.7% agarose gel and the concentration was also checked by Qubit fluorometer 3.0 (Invitrogen, Thermo Fisher Scientific, Singapore). Isolated DNA was amplified using 16S rRNA primers (27F‐5′AGAGTTTGATCTGGCTCAG3′ and 1492R‐5′ TACGGTACCTTGTTACGACTT 3′). The 16S rRNA gene sequencing was carried out using an ABI 3730xl sequencer (Applied Biosystems). Then, the phylogenetic tree was constructed using Molecular Evolutionary Genetics Analysis (MEGAX software, latest version 11) with selection of maximum likelihood at the Kimura 2‐parameter model and the aligned data was bootstrapped 1000 times for a high level of certainty for topology confidence of the tree. The genome sequence of *Escherichia coli* K‐12 (EG10823) was used as an outgroup to root the tree.

#### 2.9.3 Nucleotide Sequence Accession Numbers

The 16S rRNA gene sequence of the *Enterobacter* sp. DSAAI‐4 isolate was deposited in GenBank under the accession number PQ898114.

#### 2.9.4 Statistical Analysis

Experiments were performed in triplicate and means ± standard deviation (SD) were assessed using one‐way ANOVA and Fisher's least significant difference (LSD) Post Hoc Multiple Comparisons for more than two means, using IBM SPSS Statistics 27 (IBM Corp., Armonk, NY, USA). For culture conditions, Origin 2018 version was used. Design Expert (version 13) was used for RSM‐based optimisation. Data differences in means were considered statistically significant at a level of *p* < 0.05.

## 3 Results

### 3.1 Morphological and Biochemical Characteristics of Electrogenic Bacteria

It was observed that black‐coloured MnO_2_ supplemented chromogenic media was changed due to biofilm formation of EBIs (Figure 2b). Among the biofilm samples taken from the respective MFC anode electrodes of the study samples, 17 EBIs (23.9%) were obtained from brewery‐ activated sludge, 8 EBIs (11.3%) from brewery wastewater, 7 EBIs (9.9%) from domestic wastewater sludge, 7 EBIs (9.9%) from domestic effluent wastewater, 8 EBIs (11.3%) from sugar factory wastewater, 12 EBIs (15.5%) obtained from textile wastewater and 13 EBIs (18.3%) were recorded from the soil sample. Out of the total 72 EBIs, based on their biofilm‐forming potential, 26 EBIs were further characterised. These EBIs exhibited various features (Supporting Information: Table A_3_). Among the 26 electrogenic isolates analysed, 14 (53.8%) were identified as Gram‐positive and 12 (46.2%) were Gram‐negative EBIs (Supporting Information: Fig. A_4_). It was also noted that 18 (69.23%) EBIs were motile. Furthermore, as indicated with representative isolates from Supporting Information: Fig. A_5_, 8 (30.8%) of EBIs were citrate positive, 6 (23.1%) were found to be indole positive, all EBIs had shown positive results for catalase, 19 (73.1%) were urease positive, 4 (13.4%) were positive for H_2_S production, 16 (61.2%) were positive for the oxidase test, 12 (46.2%) were methyl red positive and 12 (46.2%) positive for the VP test.

### 3.2 Extracellular Enzyme Activities (EEA) of EBIs

**FIGURE 1:**
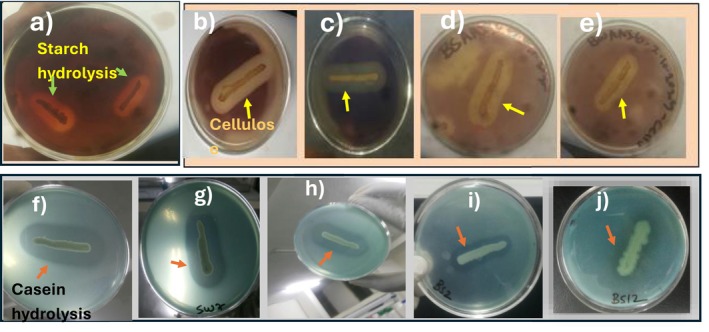
Extracellular enzyme producing selected electrogenic isolates with clearance zones. (a) Shows amylase‐producing EB‐ DSAAI‐4 with clearing zones formed around the spotted colony. A zone of inhibition was observed after a 0.1% (w/v) iodine solution had been flooded. (b–e) Cellulase producing EBIS with designated letters, a (DSAAI‐4), b (SFWWI‐7), c (BSAAI‐16), d (BWAAI‐6). CMC agar plates showed positive results with clearing zones in the presence of an indicator 0.1% (w/v) iodine solution. (f–j) shows the best selected electrogenic isolates producing protease enzyme with clearing zones around colonies, including BCG‐skimmed milk agar plates f (DSAAI‐4), g (SFWWI‐7), h (SSWI‐1), i (BSAAI‐2) and j (BSAAI‐12).

Enzymatic hydrolysis of starch (1%, w/v) by amylase was revealed by 7 EBIs (26.9%) having clear zones (Figure 1a) around their colonies when it was flooded with Gram's iodine (0.1%, w/v). Similarly, certain EBIs exhibited proteolytic activity with 11 (42.3%) isolates, which showed anionic BCG dye binding capabilities. Protease activity was observed with clear zones formed around grown colonies of EBIs (Figure 1f–j). Cellulase‐producing EBIs were detected in the presence of Carboxymethylcellulose (CMC) with clear zones after NA was flooded with Gram's iodine (0.1%, w/v) (Figure 1b–e). Accordingly, 10 (38.5%) EBIs were cellulase positive by forming clear zones after 48 h incubation.

### 3.3 Bio‐Electrochemically Relevant In Vitro Biofilm Formation

**FIGURE 2:**
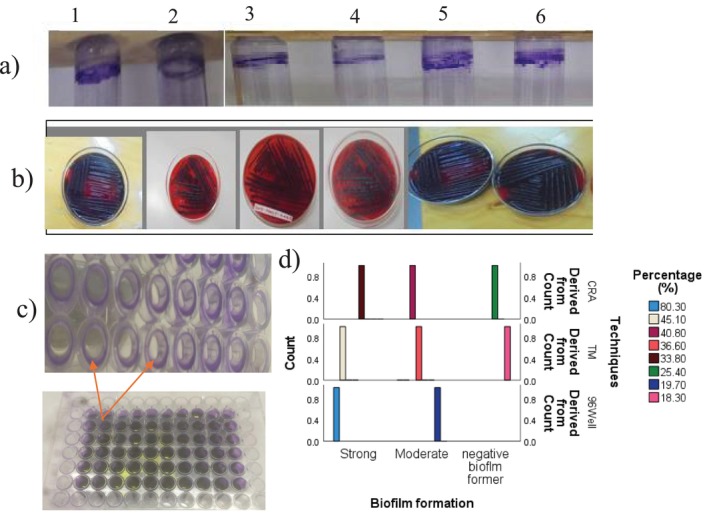
Screening through visualisation and quantification of biofilm formation for electrogenic isolates. (a) Images showing test tube method for biofilm formation test using 0.1% (w/v) crystal violet, (b) Shows for plate technique, qualitative Congo red agar method biofilm visualisation test and colonies with black colour are positive for biofilm formation, (a, b) 1, 2, 3, 4, 5, 6 represents for EBIs of which DSAAI‐4, BSAAI‐7, BWAAI‐1, DSAAI‐5, SFWWI‐7 and TXAI‐10 biofilm layers and dry crystalline that was strongly developed, respectively. (c) Shows 96 well microtiter plate method biofilm investigation after crystal violet staining and dissolving with 33% glacial acetic acid (v/v), (d) screening potential biofilm forming EBIs based on standard optical cutoff values as strong biofilm formers, moderate and weak biofilm formers and where only strong biofilm formers that satisfied in all methods were taken for further biochemical and enzymatic analysis, in this case 26 isolates showed strong biofilm formation using the three methods.

Among the total isolates tested for CRA, 25 EBIs (33.8%) were strongly positive biofilm formers with black crystalline agar plate colonies (Figure 2b, 1–6), 40.8% of isolates were moderately positive with black colonies but not dry crystalline and 25.4% of isolates were found to be non‐biofilm formers. In addition, the occurrence of visible biofilm after Crystal violet method was examined (Figure 2). Furthermore, a qualitative assay was also studied by measuring OD_570nm_. Maximum biofilm production was examined with the value of 1.218 ± 0.109777 nm and the minimum was 0.621 ± 0.047843 nm at OD_570nm_ (Supporting Information: Table A_4_). From the tested isolates, 45.1% strongly formed a visible line of film, 19.7% were moderately forming a biofilm. Nonetheless, despite some in vitro phenotypic tube methods showing differences in visible film layers (Figure 2a, 1–6), OD_570nm_ based detection through dissolving with acetic acid (33%, v/v) resulted in biofilm formation for all isolates using OD cut‐off index categories. Furthermore, among the total tested isolates using the microplate method (Figure 2c), the potential for biofilm formation range was grouped with cut‐off values. Interestingly, this method resulted in 80.3% strong biofilm formers with OD categories 4 × ODc < OD and 19.7% moderate biofilm formers with cut‐off categories OD ≤ 4 × ODc. Maximum and minimum biofilm quantity with absorbance read at OD_570nm_ were 1.4955 ± 0.18592 and 0.625667 ± 0.076631, respectively (Supporting Information: Table A_4_).

**FIGURE 3:**
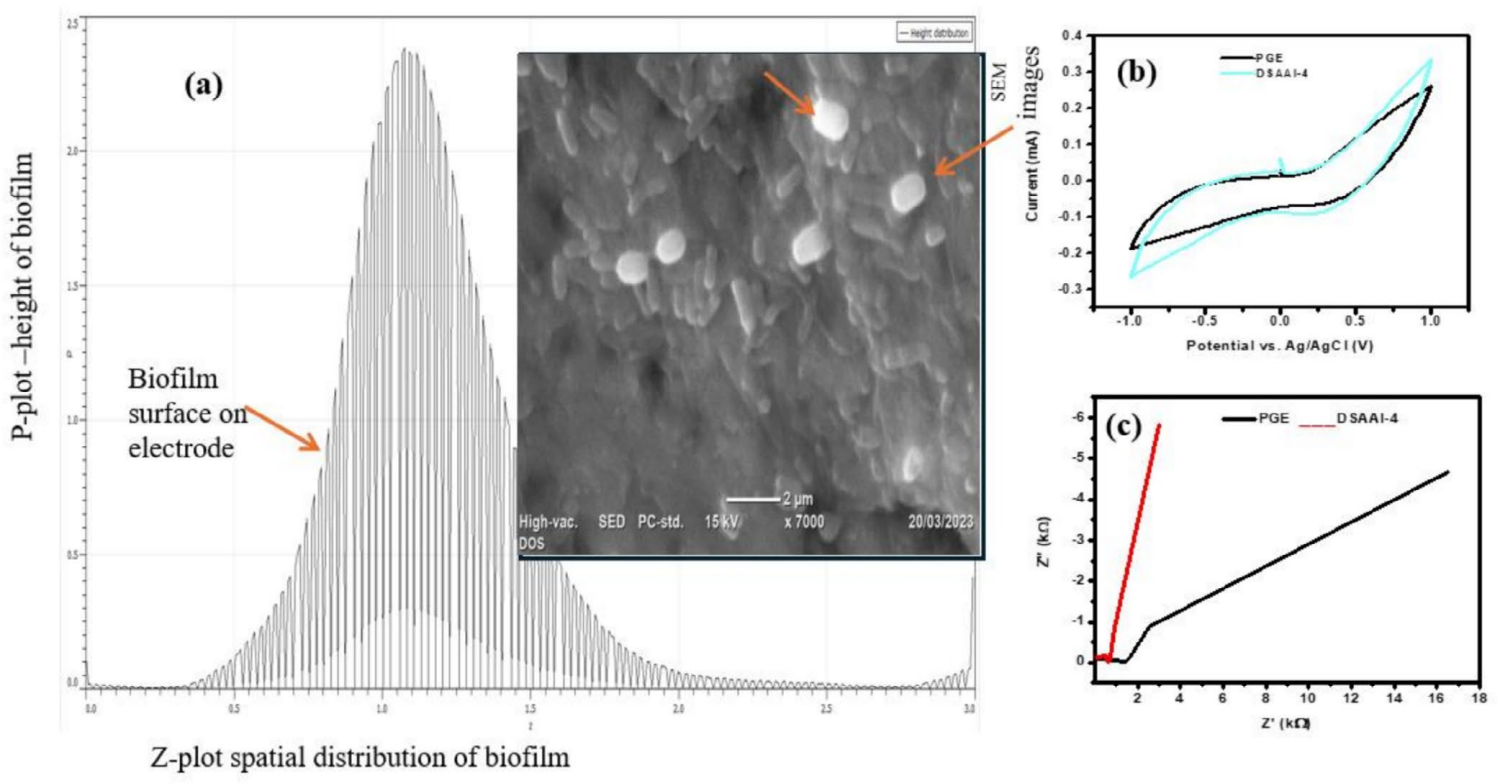
Cross‐section analysis and electrogenic properties of DSAAI‐4; (a) Depicting p–z plot showing biofilm height and distribution on the pencil graphite electrode. SEM images reveal good biofilm formation on the electrode; (b) Electrogenic properties of an isolate DSAAI‐4 was shown using cyclic voltammetry (CV) analysis in the potential window ranging from −1 to +1 V at a scan rate of 50 mV s^−1^, where areas under the CV curves for DSAAI‐4 was larger than the bare PGE that confirms a good potential with electrogenic properties; (c) Electrochemical impedance spectroscopy (EIS) analysis in the frequency range of 100 kHz to 10 mHz with 10 mV amplitude showing best electrical conductivity with less resistance for DSAAI‐4 compared to bare PGE.

As it was depicted in Figure 3a, the surface morphology of accumulated biofilm on the PGE anode was observed using scanning electron microscopy (SEM). There were short rod‐shaped cylindrical greyish‐white bacterial cells forming chains in the biofilm that was developed on the PGE, along with other straight pili‐like structures that allowed for signalling and cellular interactions. In addition, as shown in Figure 3b, validation of electrogenic behaviour and biocatalyst activity for the isolate DSAAI‐4 showed redox activity during both forward and reverse scans. *Enterobacter* sp. strain DSAAI‐4 oxidised organic materials in the anode chamber to produce a maximum voltage of 1.93 ± 0.31 mV in MFC. Additionally, as illustrated in Figure 3b, the CV peaks demonstrated the ability of our current EBI to transfer an electron to the anode electrode with oxidation and redox peaks of 0.032 and −0.0998 mA, respectively, with a potential window from −1 to +1 V at a scan rate of 50 mV/s.

### 3.4 Cultivation Conditions on Commercial Carbon Sources

**FIGURE 4:**
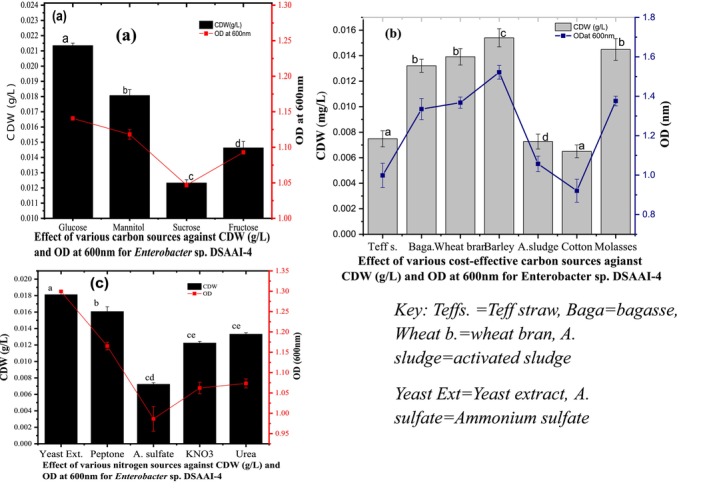
Schematic representations on the effect of nutrient types on relative growth condition for *Enterobacter* sp. DSAAI‐4, (a) shows for various commercial carbon sources with OD_600nm_ and CDW (g/L) and the result showed statistically significant variation at 95% confidence interval, (b) cultivation condition of DSAAI‐4 against with the different agro‐industrial waste and activated sludge as alternative and cost‐effective carbon source with OD_600nm_ and CDW (g/L), where barley bran showed best growth condition, (c) effect of the growth against various nitrogen sources with analysed growth yields in terms of OD_600nm_ and CDW (g/L). Data represents standard deviation (SD) for triplicates of CDW (g/L) and OD_600nm_. Means were compared using least significant difference (LSD) Post hoc tests (*p* < 0.05) of homogeneous subset for alpha showed with different small letters on the column bar for pairwise comparisons.

In vitro cultivation conditions for a potent electrogenic isolate, *Enterobacter* sp. DSAAI‐4 was examined using various commercial carbon sources. The isolate showed maximum cell growth OD_600nm_ (1.1407 ± 0.00316) value and residual biomass expressed as cell dry weight (CDW) (0.02135 ± 0.00152 g/L) when glucose (1%, w/v) was used as a sole carbon source. Contrary to this, the lowest OD_600nm_ (1.04689 ± 0.00522) and CDW (g/L) (0.01233 ± 0.0006) resulted against sucrose for the *Enterobacter* sp. DSAAI‐4 (Figure 4a). In the recent study, it was confirmed that significant variations were detected between glucose and fructose (*p* < 0.001), glucose and mannitol (*p* < 0.001), glucose and sucrose (*p* < 0.001), fructose and mannitol (*p* < 0.001), fructose and sucrose (*p* < 0.001) and sucrose and mannitol (*p* < 0.001) against the mean value of OD_600nm_ at multiple comparisons test using one‐way ANOVA for *Enterobacter* sp. DSAAI‐4 growth (Supporting Information: TableA_7_). Similarly, variation was noted between glucose and fructose (*p* < 0.001), glucose and mannitol (*p* = 0.033), glucose and sucrose (*p* < 0.001), fructose and mannitol (*p* = 0.002), mannitol and sucrose (*p* = 0.014) and fructose and sucrose (*p* = 0.021) CDW (g/L) using one‐way ANOVA against *Enterobacter* sp. DSAAI‐4 (Supporting Information: Table A_8_).

### 3.5 Nitrogen Sources Against the Growth of *Enterobacter* sp. DSAAI‐4

Yeast extract amended medium was found to be the best nitrogen source for the growth of *Enterobacter* sp. DSAAI‐4 with the highest OD_600nm_ (1.2995 ± 0.01238 nm) and CDW (0.01812 ± 0.000552 g/L). The lowest CDW (g/L) (0.00723 ± 0.000464 g/L) and OD_600nm_ (0.98621 ± 0.03575 nm) resulted from the use of ammonium sulfate against *Enterobacter* sp. DSAAI‐4 (Figure 4c). The one‐way ANOVA revealed that there was statistically significant variation in terms of OD_600nm_ between yeast extract and peptone (*p* < 0.001), yeast extract and ammonium sulfate (*p* < 0.001), yeast extract and potassium nitrate (*p* < 0.001), yeast extract and urea (*p* < 0.001), peptone and ammonium sulfate (*p <* 0.001), peptone and potassium nitrate (*p* < 0.001) and peptone and urea (*p* < 0.001). On the other hand, there were no statistical differences between ammonium sulfate and potassium nitrate (*p* = 0.7), ammonium sulfate and urea (*p* = 0.051) and potassium nitrate with urea (*p* = 0.098) (Figure 4c and Supporting Information: Table A_9_). For all nitrogen sources, the difference in CDW (g/L) was statistically significant (Supporting Information: Table A_10_).

### 3.6 Culture Cultivation on Alternative Cost Effective Agro‐Industrial By‐Products

Modified growth media with cheap carbon sources resulted in BB supplemented medium being obtained with the best growth for the present isolate with an OD_600nm_ of 1.52167 ± 0.03476 nm and a yield of CDW (g/L) of 0.01541 ± 0.000071 g/L, followed by activated sludge with an OD_600nm_ of 1.37603 ± 0.02453 nm (Figure 4b). In addition, alternative nutritional sources, molasses, bagasse and wheat bran resulted in a growth of OD_600nm_ of 1.3676 ± 0.02828, 1.33493 ± 0.005358 and 1.05731 ± 0.03936 nm, respectively. Low growth with selected parameters was examined when cotton waste was supplemented as a carbon source with an OD_600nm_ of 0.92061 ± 0.05839 nm and CDW (g/L) of 0.0065 ± 0.000503 g/L. Growth variations with different cost‐effective agro‐industrial waste supplements for the isolate *Enterobacter* sp. DSAAI‐4 were obtained by one‐way analysis of variance at a confidence level of 95%. Thus, statistically there was a significant difference for OD_600nm_ at the 95% level of confidence between BB with all enriched carbon sources using pairwise comparisons of the post hoc test (*p* < 0.001); however, the OD_600nm_ value difference was not statistically supported between molasses with bagasse (*p* = 0.416), molasses with activated sludge (*p* = 0.610) and activated sludge with bagasse (*p* = 0.195) (Supporting Information: Table A_11_). The yield of CDW (g/L) with BB modified medium against all utilised agro‐industrial carbon substrates showed statistically significant differences (*p* < 0.001). However, a pairwise comparison of one‐way ANOVA did not support the yield of CDW (g/L) for the present study isolate when employed between molasses and activated sludge (*p* = 0.313) and wheat bran and cotton waste (*p* = 0.236) (Supporting Information: Table A_12_).

### 3.7 Growth Condition Using Response Surface Methodology (RSM) Approach

Response surface methodology using a CCD was employed to determine the growth characteristics of *Enterobacter* sp. DSAAI‐4. The impact of temperature, pH and salt concentration and their interactive effects on culture growth conditions with previously optimised best carbon and nitrogen sources in response to OD_600nm_ and CDW (g/L) was analysed. The analysis of the CCD generated a quadratic polynomial model as a function of OD_600nm_ and CDW (g/L) using analysis of ANOVA with a total of 20 duplicated experimental runs (Table 2). Based on the ANOVA result, it was revealed that the *F* value of 68 for OD_600nm_ (Supporting Information: Table A_5_) and 34.13 for CDW (g/L) (Supporting Information: Table A_6_) implied all the significance of the model.

**TABLE 2:** Response surface central composite design model for DSAAI‐4 growth condition.

Run	Factor 1	Factor2	Factor 3	OD_600nm_		CDW (g/L)	
	A: temperature (°C)	B: pH	C: salt concentration (g/L)	Actual (non‐transform)	Predicted (non‐transform)	Actual (non‐transform)	Predicted (non‐transform)
1	35	7	0.2	0.9380	0.9717	0.0018	0.0028
2	25	11	15	0.8450	0.8543	0.0009	0.0020
3	18	7	8.5	0.9020	0.9593	0.0082	0.0078
4	52	7	8.5	1.13	1.11	0.0132	0.0120
5	35	0.3	8.5	0.9640	1.00	0.0078	0.0073
6	35	13	8.5	0.6100	0.6117	0.0009	−0.0003
7	35	7	8.5	1.41	1.42	0.0188	0.0184
8	35	7	8.5	1.464	1.42	0.0199	0.0184
9	45	3	15	1.08	1.11	0.0098	0.0110
10	25	3	2	1.12	1.11	0.0092	0.0085
11	35	7	8.5	1.46	1.42	0.0198	0.0184
12	25	11	2	0.7020	0.6483	0.00083	0.0008
13	35	7	8.5	1.41	1.42	0.0169	0.0184
14	45	11	2	0.7020	0.7311	0.0010	0.0009
15	25	3	15	1.07	1.01	0.0049	0.0061
16	35	7	19.4317	1.20	1.21	0.0108	0.0082
17	35	7	8.5	1.39	1.42	0.0178	0.0184
18	45	11	15	1.11	1.10	0.0078	0.0096
19	35	7	8.5	1.39	1.42	0.0169	0.0184
20	45	3	2	1.07	1.04	0.0057	0.0058

#### 3.7.1 Effects on OD_600nm_ and Cell Dry Weight

A potential selected electrogenic DSAAI‐4 isolate showed the highest growth condition with actual response (OD_600nm_ 1.464 ± 0.0458) at 35°C, 7 pH, and 0.12 M NaCl followed by the lowest OD_600nm_ (0.61) at the strongly basic region of pH 13. From the above‐mentioned response ranges, the predicted results for OD_600nm_ and CDW (g/L) were 1.42 and 0.0184, respectively, at optimum 35°C, pH 7 and 0.12 M of NaCl (Table 2). As shown in Supporting Information: Table A_5_, based on the ANOVA result, it was revealed that the *F* value of 68 for OD_600nm_ with only 0.01% chance that the *F* value could occur due to noise. As indicated from Supporting Information: Table A_5_, temperature as a single factor on OD_600nm_ showed statistical significance at a *p* value of 0.05 (*p* = 0.0045 with 13.25 *F* value), pH (*p* < 0.0001, with an *F* value of 84.83) and NaCl concentration (*p* = 0.0002, with 30.68 *F* value). Analysis of interactive effects from the model was found to be significant on OD_600nm_ value, which includes temperature and pH (AB) (*p* value of 0.0447), temperature and salt concentration (AC) (*p* = 0.0287) and BC (*p* = 0.001). Similarly, replicates of center points to understand the best prediction with pure error for the lack of fit of the actual data with the model predicted one also showed statistical significance (*p* < 0.0001).

**FIGURE 5:**
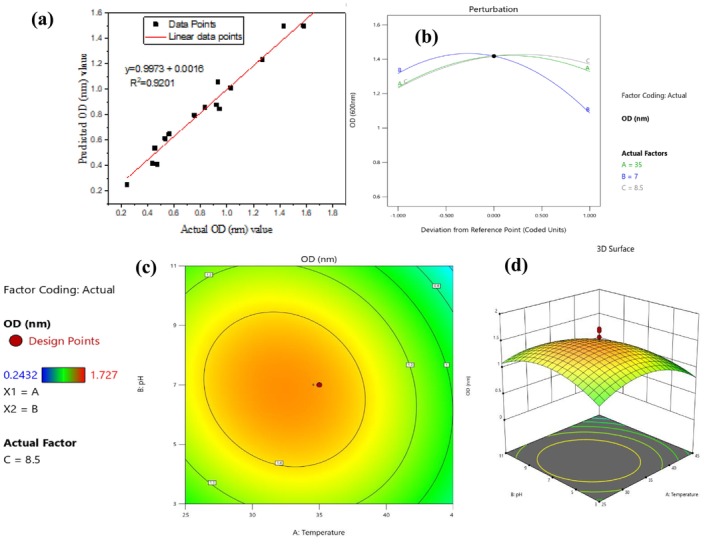
Response surface plots for the effect of designed parameters on cultivation condition: (a) plot of predicted Vs actual value for OD_600nm_ with strong linear correlation coefficient (*R* ^2^ = 0.9), (b) perturbation graph representing comparison of influence of temperature (A), pH (B) and salt concentration (C) interactive effects on culture condition in terms of OD_600nm_, (c) 2D Contour surface plot showing combined interactive effects of selected parameters on OD_600nm_ and (d) 3D surface plots showing the effect of setting parameters on cultivation condition of DSAAI‐4 using OD_600nm_ response.

In addition to *p* value, the effectiveness of the developed model was validated using parameters including coefficient of determination (*R* ^2^), adjusted *R* ^2^, predicted *R* ^2^ and coefficient of variation (CV%) for the response of OD_600nm_. Interestingly, the current finding was depicted with predicted *R* ^2^ = 0.8995 (89.95%) and *R* ^2^_adj_ = 0.9695 (96.95%) where the expected coefficient of determination was found to be close to the adjusted *R* ^2^ with an acceptable range of less than 20% and a slight standard deviation (0.0464), which was found to be a better‐predicted response (Table 3). Experimentally measured values (in this case, actual values) and predicted values have shown a straight line in normal % probability along with externally studentised residual plots (Figure 5a).

**TABLE 3:** Statistical analysis of variance (ANOVA) of the RSM model corresponding to the response.

Source	SS^a^	C.V (%)	Std. dev	DF^b^	MS^c^	*F*	*p*	Adjusted *R* ^2^	Predicted *R* ^2^
Quadratic	2.71	4.22	0.0464	3	0.9018	30.06	< 0.0001	0.9695	0.8995
Residual	0.2347			6	0.0391				
Total	0.3202			9	0.0562				

^a^ Sum of squares.

^b^ Degree of freedom.

^c^ Mean square.

Furthermore, the obtained 2.6% for the Lack of Fit *F* value indicates that the Lack of Fit is not significant compared to the pure error and the model supports a non‐significant lack of fit as the best design (Supporting Information: Table A_5_). Different diagnostic plots were assessed to examine model validity for the following experimental activities using predicted optimised conditions settings. Typical plots of residuals with the function of normal % probability regression and predicted versus actual (Figure 5a) were among the quadratic model validation graphs. In this respect, as shown in Table 3, low values of the coefficient of variation (CV = 4.22%) were obtained and the validity of the model for further in vitro activities is strongly recommended.

The predicted *R* ^2^ of 0.8995 showed reasonable agreement with the adjusted *R* ^2^ of 0.9695; that is, the difference was less than 0.2. Interactive effects of all study variables were simultaneously analysed using the perturbation graph (Figure 5b) to see the effect on OD_600nm_ of *Enterobacter* sp. DSAAI‐4. Although all factors on the perturbation plot showed deviations on either side of the center point, a higher constant OD_600nm_ value was obtained at the coded zero level of the design. From the perturbation plot, the effect of pH on OD_600nm_ was analysed and as pH increases after the center point, the OD_600nm_ value starts to drop. Flat plots for temperature (A) and salt concentration (C) suggested that these variables have little effect on OD_600nm_ value.

Similarly, by inspection of the plot from Supporting Information: Fig. A_6a_, CDW (g/L) was examined using a perturbation plot and as the input variable, pH increase after the center points influenced CDW value to decrease. Second‐order model analysis using 2D counter graphs and 3D surface plots was conducted. Accordingly, 2D circular counter graphs showed significant interaction for an increase of OD_600nm_ with combined interactive effects of temperature between 35°C and 40°C and pH in between 5 and 7 (Figure 5c).

Quantitative assessment of cell biomass using the combined effects of factors was analysed and the highest biomass was 0.0199 ± 0.001 g/L at 35°C, 7 pH, and 0.12 M NaCl, followed by the lowest OD_600nm_ (0.00083 ± 0.00006 g/L) at the pH region of 11°C and 25°C. More briefly, based on ANOVA analysis, factors that significantly contributed to the CDW (g/L) were temperature (*p* = 0.0217), pH (*p* = 0.0006) and salt concentration (*p* = 0.0055) (Supporting Information: Table A_6_). ANOVA for the quadratic model revealed that, the non‐significant lack of fit for CDW (g/L) with an *F* value of 2.05 relative to pure error (0.00185%) was acceptable. The outcome of the RSM model resulted in that predicted coefficient of determination (*R* ^2^) and adjusted *R* ^2^ with values of 0.8240 (82.4%) and 0.9401 (94.01%), respectively. It was shown that the CDW (g/L) value proportionally goes with that of OD_600nm_ based on analysis of interactive effects of perturbation plots. In this case, as the pH range increases, decreasing CDW (g/L) was observed. Both temperature and salt concentration have slight effects from the reference zero points; however, based on these interactive variables, the isolate could tolerate high salt concentration (0.332 M) with 0.0108 g/L and temperature (52°C) with 0.0132 g/L of CDW.

#### 3.7.2 Experimental Validation of the Model

**FIGURE 6:**
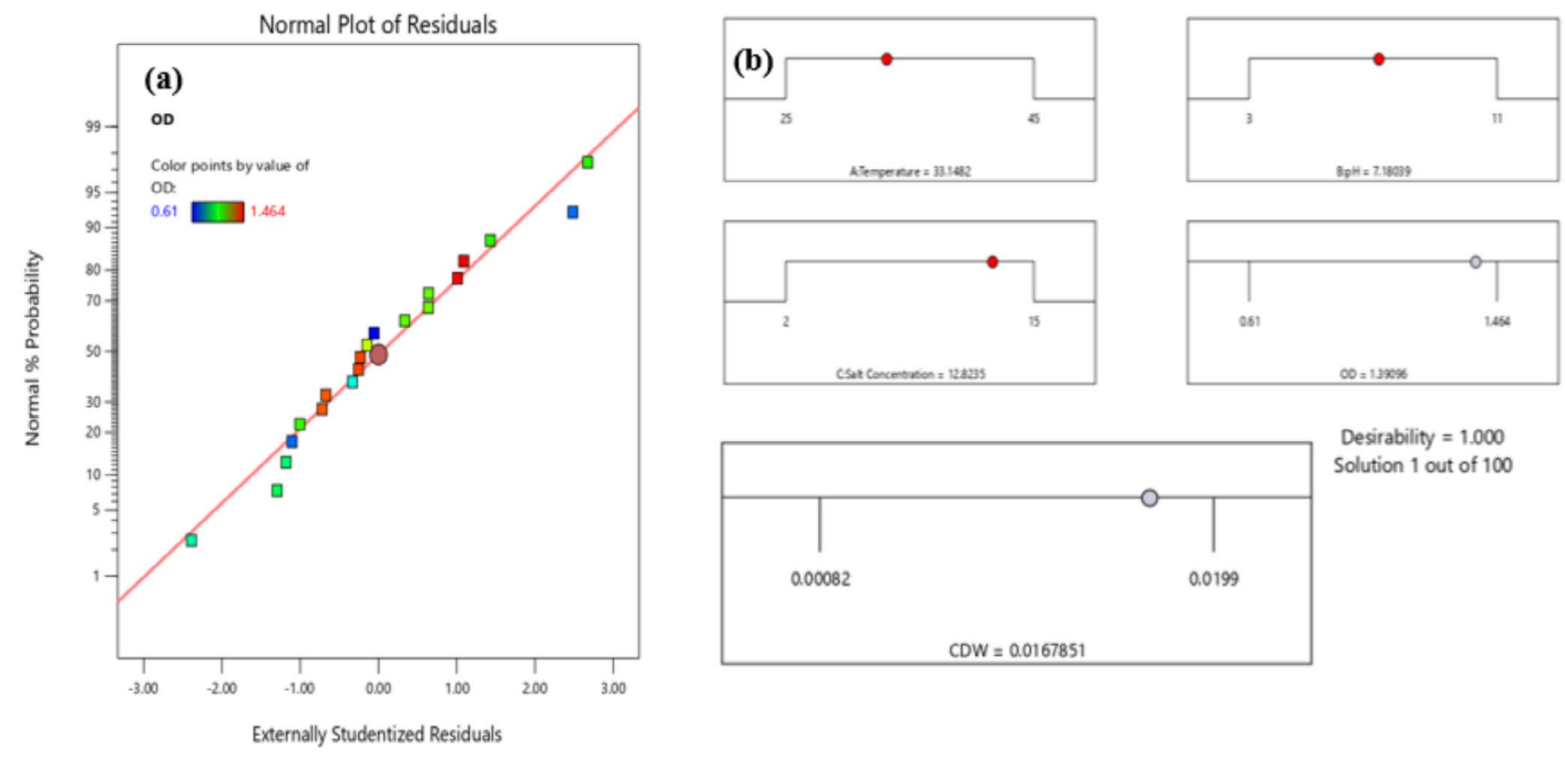
Graphical plot shows for numerical optimization of process variables; (a) Normal residual plot that shows linear spread of data points which validates for strong relationship of designed model; (b) ramp plot of optimization solution for the responses, red dots on each ramp shows suggested optimum factor settings and the dot with grey colour reflects model predicted responses for OD_600nm_ and CDW (g/L).

To demonstrate the performance of optimal values derived from the quadratic model for the factors, a triplicate‐based experiment was performed for further validation of the predicted response. In this case, our study isolate was cultivated with suggested optimal conditions (Figure 6b); thus, 1.368 ± 0.00954 OD_600nm_ values and 0.0117 ± 0.007769 of CDW (g/L) were obtained under controlled optimal conditions. Interestingly, the model predicted values for OD_600nm_ and CDW were 1.39096 and 0.0168, respectively, under the selected optimum designed factor. The difference from the experimentally obtained value for OD_600nm_ and CDW (g/L) was 0.02296 (2.3%) and 0.0051 (0.5%), respectively. The obtained values between the identified quadratic model, the proposed optimum condition and experimental value showed much closer agreement for the condition of culture growth.

### 3.8 EBIs Profile Using Biotyper MALDI‐TOF MS

Based on the MALDI‐TOF MS analysis, 24 (92.31%) out of 26 isolates were successfully identified with different score values. Among these, 16 had a logarithmic score value (LSV) of ≥ 2.0, 8 had LSV of ≥ 1.7 and 2 (7.69%) had an LSV of < 1.7, which was deemed unreliable according to the database (Table 4). Based on MALDI‐TOF MS profiling, the dominant EBIs were found to be *Bacillus* (42.31%) and *Pseudomonas* (23.1%). The remaining EBIs were *Aeromonas*, *Brevundimonas*, *Citrobacter, Delftiaacid*, *Escherichia*, *Kocuria*, and *Roultella* genera (Table 4). MALDI‐TOF MS fingerprint analysis assigned DSAAI‐4 to the genus level, *Pseudomonas* species (LSV = 1.71) based on mass‐to‐charge ratios (m/z) of protein profiles and its potential electrogenic properties. Further analysis for isolate DSAAI‐4 was investigated using 16S rRNA gene sequencing analysis for species identification.

**TABLE 4:** Species of bacteria isolated from anode biofilm using MALDI‐TOF MS (Zybio EXS3000 Zybio Inc., China).

EBIs from anode region	Predicted EBIs based on LSV	LSV	Designated EBIs	EBIs from anode region	Predicted EBIs based on LSV	LSV	Designated EBIs
BSAAI‐2	*Bacillus cereus*	2.07	*B. cereus* BSAAI‐2	DSAAI‐1	*Bacillus cereus*	2.48	*B.cereus* DSAAI‐1
BSAAI‐7	*Pseudomonas*	1.75	*Pseudomonas* sp. BSAAI‐7	DSAAI‐4	*Pseudomonas*	1.71	*Pseudomonas* sp. DSAAI‐4
BSAAI‐8	*Bacillus*	1.86	*Bacillus* sp. BSAAI‐8	DSAAI‐5	*Kocuria marina*	2.18	*K. marina* DSAAI‐5
BSAAI‐12	*Bacillus*	1.86	*Bacillus* sp. BSAAI‐12	DWASTUI‐3	*Escherichia coli*	2.42	*E. coli* DWASTUI‐3
BSAAI‐3	*Bacillus cereus*	2.23	*B. cereus* BSAAI‐3	DWASTUI‐4	*Bacillus cereus*	2.34	*B. cereus* DWASTUI‐4
BSAAI‐16	*Brevundimonas diminuta*	2.14	*B. diminuta* BSAAI‐16	DWASTUI‐6	*Delftia acidovorans*	2.48	*D. acidovorans* DWASTUI‐6
BSAAI‐17	*Pseudomonas*	1.71	*Pseudomonas* sp. BSAAI‐17	DWASTUI‐7	*Unreliable*	1.62	*NA*
BWAAI‐1	*Bacillus cereus*	2.07	*B. cereus* BWAAI‐1	SFWWI‐7	*Bacillus cereus*	2.18	*B. cereus* SFWWI‐7
BWAAI‐2	*Pseudomonas*	1.76	*Pseudomonas* sp. BWAAI‐2	TXAI‐3	*Bacillus cereus*	2.31	*B. cereus TXAI‐3*
BWAAI‐4	*Pseudomonas*	1.71	*Pseudomonas* sp. BWAAI‐4	TXAI‐4	*Bacillus cereus*	2.44	*B. cereus* TXAI‐4
BWAAI‐5	*Bacillus subtilis*	2.23	*B. subtilis* BWAAI‐5	TXAI‐10	*Pseudomonas*	1.98	*Pseudomonas* sp. TXAI‐10
BWAAI‐8	*Aeromonas veronii*	2.12	*A. veronii* BWAAI‐8	SSWI‐1	*Citrobacter freundii*	2.24	*C. freundii* SSWI‐1
BWAAI‐6	Unreliable	1.45	NA	SSWI‐9	*Roultella ornithinolytica*	2.45	*R. ornithinolytica* SSWI‐9

### 3.9 16S rRNA Gene Analysis for EBIs

**FIGURE 7:**
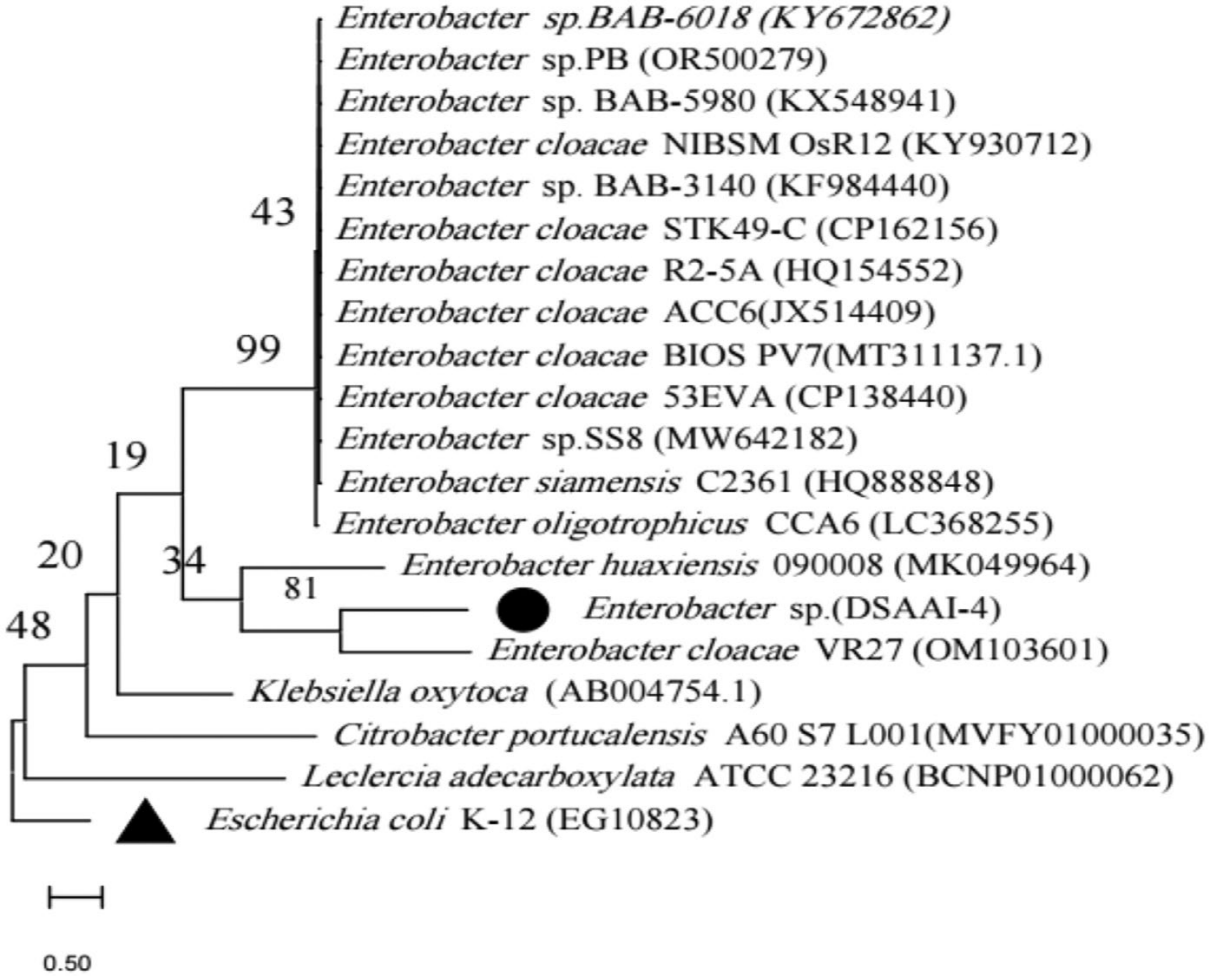
Evolutionary relationships with identified EBI taxa. The evolutionary history was inferred using the Maximum Likelihood method and kimura 2‐parameter model (Kimura 1980). The tree with the highest log likelihood is shown. The percentage of trees in which the associated taxa clustered together in the bootstrap test (1000 replicates) is shown next to the branches. Initial tree(s) for the heuristic search were obtained automatically by applying Neighbour‐Join and BioNJ algorithms to a matrix of pairwise distances estimated using the Maximum Composite Likelihood (MCL) approach and then selecting the topology with superior log likelihood value. The tree is drawn to scale, with branch lengths measured in the number of substitutions per site. This analysis involved 20 nucleotide sequences. All ambiguous positions were removed for each sequence pair (pairwise deletion option). Codon positions included were 1st + 2nd + 3rd + Noncoding. There were a total of 1655 positions in the final dataset. Evolutionary analyses were conducted in MEGA11 (Tamura et al. 2021).

Phylogenetic analysis designated our new isolate as *Enterobacter* sp. DSAAI‐4 with 98.81% sequence similarity. The basic local alignment search tool (BLAST) and EzBioCloud homology search revealed that the strain was located in the position occupied by the genus *Enterobacter*. As depicted in Figure 7, the phylogenetic tree for the currently designated strain formed a clear phylogenetic category with different cutoff indexes; for example, 81% with *Enterobacter cloacae VR27*, 99% with *Enterobacter cloacae* strain STK49‐C and below the 50% cutoff value with *Klebsiella oxytoca, Citrobacter portucalensis and Leclercia adecarboxylata*. Currently identified EBIs showed 55.84% GC content (Table 5). The outgroup was represented by *Escherichia coli* K‐12.

**TABLE 5:** Sequence similarity between presently isolated and other strains with their calculated GC (%) content.

Bacterial 16S rRNA	Strain	Accession number	GC (%)	Sequence similarity
*Enterobacter* sp.	DSAAI‐4	PQ898114	55.84	The recent EBI
*E. oligotrophicus*	*CCA6*	LC368255	55.76	96.69
*C. portucalensis*	A60_S7_L001	MVFY01000035	54.15	97.04
*L. adecarboxylata*	ATCC 23216	BCNP01000062	53.11	97.52
*E. huaxiensis*	090008	MK049964	54.96	96.40
*K. oxytoca*	—	AB004754	54.51	96.80
*E. cloacae*	VR27	OM103601	55.98	98.81
*Enterobacter* sp.	SS8	MW642182	56.00	98.8
*E. cloacae*	53EVA	CP138440	56.05	98.8
*E. cloacae*	BIOS PV7	MT311137	56.05	98.8
*E. cloacae*	ACC6	JX514409	56.05	98.8
*E. cloacae*	R2‐5A	HQ154552	56.05	98.8
*E. cloacae*	STK49‐C	CP162156	56.05	98.8
*Enterobacter* sp.	BAB‐3140	KF984440	56.05	98.8
*E. cloacae*	NIBSM_OsR12	KY930712	56.05	98.8
*Enterobacter* sp.	BAB‐5980	KX548941	56.05	98.8
*Enterobacter* sp.	BAB‐6018	KY672862	56.05	98.8
*Enterobacter* sp.	PB	OR500279	56.05	98.8
*E. siamensis*	C2361	HQ888848	55.14	96.45

## 4 Discussion

In this study, various electrogenic bacteria from the anode electrode developed biofilm were isolated. A black coloured chromogenic medium amended with MnO_2_ was converted to white following the growth of isolates. This could be associated with the utilisation of MnO_2_ from the growth medium as an electron acceptor during their metabolic process. Briefly, based on the change of medium colour (Supporting Information: Fig. A_3_), it could be suggested that the transfer of metabolic electrons from electrogenic bacteria following substrate oxidation was facilitated by MnO_2_ as an electron acceptor. These properties of growth and decolorisation capabilities of electrogenic bacteria on medium containing MnO_2_ were similar to a previous report (Nazeer and Fernando 2022). Furthermore, the tested isolates were catalase positive, which means they metabolically release superoxide radicals to make reaction contact in their outer cell membrane and permit cells to grow in the presence of MnO_2_. Studies support our finding, discussing that electrogenic bacteria, including *S. oneidensis* MR‐1 have strategies in oxidation reactions to metal‐containing minerals with the help of the cytoplasmic membrane and structural proteins from the inner membrane (Hu et al. 2021). In addition, the application of manganese‐oxidising *Ramlibacter lithotrophicus* bacteria in wastewater treatment and organic pollutant degradation was reported (Mo et al. 2024).

Native electrogenic isolates of bacteria were screened from various industrial and domestic wastewaters using MFCs. Among the waste types, brewery wastewater was the highest hosting environment with 17 EBIs, which could be associated with the existence of more suitable biodegradable organic matter content. Real brewery wastewater as feed was utilised in a microbial electrochemical reactor and various co‐existing potential electrogenic bacterial communities were reported (Wang et al. 2016). Isolation of bacteria with electrogenic potential from the anode‐developed biofilm of different waste types demonstrates that electrogenic bacteria could be isolated from all ecosystems. Interestingly, Bond and Lovley (2003) reported that bacteria developing a biofilm on the anode surface could be mainly for electricity generation.

The appearance of an oxidation peak (0.032 mA) with a potential window from −1 to +1 V at a scan rate of 50 mV/s could indicate that the isolate contains metabolites that are playing a role in the oxidation process and electron shuttling. Comparably, earlier research employing *E. cloacae* as a biocatalyst documented its electrochemical behaviour with metabolic activity and an oxidation peak at a potential of 0.35 V (Nimje et al. 2011). The highest redox peak current (−0.0998 mA) suggests its potential role as a biocatalyst, attributed to the mediator metabolites that facilitate the transfer of electrons to the anode surface. In line with our findings, Feng et al. (2014) used a glassy carbon electrode and found that *Enterobacter* sp. strain R2B1 produced oxidation peaks at 0.290 V and redox peaks at −0.206 V in their CV data. Despite not using a single culture, Sarmin et al. (2019) reported that employing anaerobic sludge as an inoculum led to an increase in the redox peak current, which is associated with the biofilm and its mediators. Furthermore, the positive potential of the cyclic voltammogram, which indicates the presence of oxidised organic matter, can also be explained by the EBI DSAAI‐4's improved performance, which suggests that it is engaged in metabolic activities in the anodic chamber. The area under the CV voltammogram with DSAAI‐4 showed better performance than the bare PGE electrode without bacteria, which could be related to the charge storage that provides better insights with our current isolated EBI. The EBI DSAAI‐4 (Figure 3b) had a larger region beneath the CV voltammogram than the bacterial‐free PGE electrode. In addition to facilitation of charge transfer, the bigger area of this curve indicates the importance of our isolate as a possible electroactive species.

The Nyquist diagram (Figure 3c) was employed for a confirmation investigation using electrochemical impedance. The results indicated that EBI‐DSAAI‐4 exhibited superior electrical conductivity due to its lower electrical resistance when compared to the bare electrode, PGE. This finding aligns with observations made in the CV curve. The EIS spectra (Figure 3c) show that this dominance is caused by metabolic processes that contribute to the release of electrons, which in turn indicates higher electrical conductivity due to reduced electrical resistance. The EIS spectra in the presence of *Enterobacter* sp. DSAAI‐4 showed a much lower ohmic resistance (0.58 Ω) compared with bare PGE (1.45 Ω), suggesting isolate DSAAI‐4 showed its potential electrochemical properties. In accordance with our analysis, Ai et al. (2020) noted that a MFC inoculated with Pseudomonas sp. E8 exhibited significantly lower ohmic resistance and enhanced charge transfer capabilities. Furthermore, the scanning electron microscopy (SEM) image of the electrochemically active thick biofilm (Figure 3a) on the electrode surface indicates reduced resistance for charge transfer.

In the present study 69.23% of isolates demonstrated motility, which could have a potent role in mobilising organic matter for coexisting nonmotile groups and an easy degradation process. In addition, their motility could assist in colonising the anode electrode and forming aggregates of biofilm that enable the MFC reactor system to be more efficient. Similarly, it was evidenced that motile microorganisms contribute to transporting organic pollutants to enhance collaborative degradation of contaminants (McBride 2001). A phenotypic assay for citrate utilisation showed that 30.8% of the total isolates could be associated with citrate metabolism as a carbon source. More interestingly, metabolic conversion of citrate by electrogenic isolates could have a positive implication in their resistance to acidic stress, which could be used as the best metabolic process to form a robust biofilm biocatalyst to stabilise and enhance the performance of MFC systems. In line with the current findings, previous studies illustrated that most electrogenic bacteria can metabolise citrate; for example, *Citrobacter freundii* LZ‐1 produces electricity through metabolising citrate as a carbon source (Zhou et al. 2017). From this study, in vitro cultivation demonstrated few indole‐positive isolates (23.1%) that can produce indole as a byproduct, which could have a role in the electron transfer mechanism. The role of indole as a signalling molecule and facilitator in energy production by bacteria was reported (Chimerel et al. 2013). Isolation of urease‐positive isolates would have suggested a promising role in the degradation of nitrogen‐rich wastes.

Analysing enzymatic profiles of electrogenic bacteria is very crucial for their potential roles in efficient biocatalytic performance. Proteolytic activity of the currently investigated isolates showed positive results with a clear zone of inhibition around colonies. Potential isolates (42.3%) showed protease‐secreting ability, which could have contributed to facilitating degradation of organic wastewater. Similarly, Schneider et al. (2023) reported that 70% of electrogenic isolates from mud samples showed proteolysis. The study for proteolytic activity was performed using skim milk substrate as a model for protein‐rich organic matter and those showing a clear zone were degrading peptide bonds found in the skim milk substrate with the help of the protease enzyme. Thus, during the cleaving process, bromocresol green (BCG) played a great role as a pH indicator and observation of the clearance zone demonstrates the loss of intact protein content in the skim milk agar plate (Figure 1). Even though not adequately explored, isolates with protease production could have a significant contribution in maintaining anode electrode biofilm through breaking down old and allowing new biofilm formation and self‐cleaning purposes, as it was revealed from scanning images with whitish and grey colours (Figure 3a). The role of protease enzymes isolated from *B. vietnamensis* on corrosion prevention of copper‐based electrodes was reported (Moradi et al. 2019).

Hence, the current EBIs were initially isolated from the anode biofilm. The ability of the biofilm to form for facilitation of electron transfer and to stably survive harsh environments in MFCs, biofilm formation analysis was a very key analysis. In the present study qualitative observation of the CRA method biofilm assay was performed and it was found that 33.8% of EBIs were strong biofilm formers. In a study by Akbar et al. (2023) quinol oxidase‐expressing *S. epidermidis* showing electrogenic properties resulted with the CRA method for strong biofilm formation (6%), moderate biofilm formers (12%) and weak biofilm (72%). Interestingly, organic pollutant Congo red decolorisation following biofilm formation could be induced by binding of Congo red dye with bacterial secreted exopolysaccharide (EPS) molecules. Previous reports demonstrated that electroactive *Bacillus* sp. was characterised by decolorizing Congo red dye (Gomaa et al. 2021). Despite the CRA method showing strong biofilm formation, however, the tube method biofilm study demonstrated more sensitivity for strong biofilm formation (45.1%). In addition, weak biofilm formation was detected in the case of the tube method with 18.3%, but there was no statistical variation with the CRA method (*p* = 0.999). The study found that the 96‐well plate method was the best for sensitivity detection of biofilm (80.3%) formation compared with the two methods discussed above (Figure 2d). Higher biofilm detection could be linked with the contribution of crystal violet dye that localised the adherent biofilm cells (Dassanayake et al. 2020).

Determination of the optimal carbon source for the growth conditions of *Enterobacter* sp. DSAAI‐4 was studied using MSM media. The in vitro cultivation results showed that glucose as the sole carbon source had the highest CDW (g/L) and OD_600nm_ among the commercial carbon source sugars. Similarly, suitable growth conditions for electrogenic bacteria, including *Shewanella oneidensis* MR1 (Nazeer and Fernando 2022), *E. faecium* FAIR‐E 198 (Vaningelgem et al. 2006) and *S. marisflavi* BBL25 (Gurav et al. 2020) using glucose as the sole carbon source were reported. This isolate exhibited the highest CDW (g/L) of 0.0239996 ± 0.001528 and an OD_600nm_ value of 1.1407 ± 0.00316 when glucose was the sole carbon source, which could be associated with its low molecular weight that gives an advantage of being easily metabolised in addition to probably having a glucose transporter phosphotransferase system (PTS) for uptake. In contrast to our findings, sucrose promoted the best growth condition for *Bacillus tequilensis* (Murniasih et al. 2024). The lowest growth (CDW (g/L) (0.01233 ± 0.0006)) and (OD_600nm_, 1.04689 ± 0.00522) conditions were demonstrated when sucrose was employed as a sole carbon source. In line with the present study, Pylak et al. (2021) described that sucrose as a sole carbon source resulted in the lowest growth for *Pseudomonas* sp. B37/18. However, even though statistical variation with the best growth conditions was observed among different carbon sources, the current isolate, *Enterobacter* sp. DSAAI‐4 exhibited broad substrate range utilisation ability.

Interestingly, all organic and inorganic nitrogen sources employed in our study supported bacterial growth; nevertheless, variations with OD_600nm_ and cell biomass were observed. Yeast extract was a better and more efficient nitrogen source for *Enterobacter* sp. DSAAI‐4, with the highest OD_600nm_ value of 1.2995 and CDW (g/L) of 0.01812. The significant contribution of yeast extract to growth conditions for *E. faecium* F58 (Zanzan et al. 2024) and *E. casseliflavus* (Luo et al. 2022) was reported. Similar reports were given by Koim‐Puchowska et al. (2021) for the highest biomass yield of *B. subtilis* natto BS19 when yeast extract‐supplemented media were utilised. Efficient growth conditions of electrogenic bacteria *Desulfuromonas versatilis* sp. using 0.1% of yeast extract were reported (Xie et al. 2021). Contrary to the current analysis, Murniasih et al. (2024) discussed that peptone as a sole nitrogen source resulted in better growth for *B. tequilensis*. Low growth conditions were detected using ammonium sulfate as a sole nitrogen source, which agreed with the previous studies stating that ammonium alone does not generate high current and optimum growth unless nitrate or nitrite is supplemented (He et al. 2009). Furthermore, better growth conditions for *Bacillus subtilis* ANR 88 with organic nitrogen sources and its subsequent high yield of biosurfactant were also reported (Rane et al. 2017).

Agro‐industrial residues (AIR), which are inexpensive and environmentally friendly substrates, were used for media formulation for growth support. Utilisation of agro‐industrial wastes as a carbon source for *Enterobacter* sp. DSAAI‐4 could be suggested as the first‐time report. In this regard, our study revealed that the medium supplemented with BB was preferably utilised as a carbon source and resulted in significant CDW (g/L) and OD_600nm_ (Figure 4b). Interestingly, Gurav et al. (2020) reported higher cell growth of electrogenic *Shewanella marisflavi* BBL25 when grown with barley straw. Furthermore, they compared the current output density with glucose‐fed and barley straw‐fed, with better results. In line with this, the biocatalyst potential of electrogenic bacteria in oxidising agro‐industrial waste biomass as the carbon source and the capability in converting it into electrical energy have been reported (Gurav et al. 2020). Comparable efficient growth ability also resulted in both sugarcane molasses and activated sludge as carbon sources, which could suggest that growth conditions have a remarkable association with nutrient content and efficient enzymatic potentials, which help in the waste treatment process. In alignment with our findings, Mathias et al. (2017) observed that brewery wastes are rich in organic matter. Consequently, these wastes ought to be used for creating alternative media for microbe cultivation rather than being discarded. In line with this, low‐cost sugar processing wastes and wastewater sludge were exploited as essential carbon source growth media for the efficient production of extracellular polymeric substances and their role in pollutant load reduction was reported previously (Siddeeg et al. 2020).

The OD_600nm_ value of 1.37603 ± 0.002453 nm indicates that activated sludge, used as a nutrient source, produced satisfactory culture growth. This highlights the abundant organic matter present in the sludge, which supports the growth of our isolate. Furthermore, the present isolate demonstrated enhanced growth conditions compared to previously reported conditions for *Rhizobium trifolii* using dairy sludge as a nutrient source, with an OD at OD_600nm_ of 0.683 ± 0.010 nm (Singh et al. 2013). Bagasse also demonstrated ideal growth with an OD value of 1.33493 ± 0.005358 nm, indicating that it may be used as a substrate for *Enterobacter* species. Its abundance of fermentable carbohydrates could be linked to this growth state. Similar to our analysis, bagasse is the optimum substrate for microbial enzyme development and production, according to Naik et al. (2023), who made a similar observation to ours. Interestingly, wide nutrient utilisation as a carbon source by currently identified isolates highlights their metabolic versatility and the presence of diversified genes that enable degradation of various organic compounds.

Culture conditions of bacterial growth are governed by various factors that adversely influence cell growth. This study attempted to examine in vitro growth conditions of *Enterobacter* sp. DSAAI‐4 through optimisation of pH, temperature and salt tolerance using RSM based CCD. The present work is novel in bestowing different cost‐effective carbon sources as alternative growth media and demonstrates interactions across different growth factors using statistical regression analysis and RSM models. Accordingly, interactive effects across multiple variables on cultivation conditions were studied for the first time for our isolated species using the simultaneous RSM approach and *Enterobacter* sp. DSAAI‐4 could grow from 18°C to 52°C temperature range, pH 3–11 and 0.0086–0.33 M of NaCl. In this study, OD_600nm_ values of the DSAAI‐4 isolate were influenced by all selected variables, including temperature (*p* = 0.0045), pH (*p* < 0.0001) and salt concentration (*p* = 0.0002) at the quadratic model source, which highlights that interactive effects of different variables influence growth conditions unless an optimum range that enhances the growth condition is maintained.

The value of the correlation coefficient (*R* ^2^) between the experimental response and the model predicted value was 0.8995, which could suggest that the present model is a good prediction for the experimental cultivation conditions. In agreement with our findings, values of the predicted and adjusted *R* ^2^ close to 1 are considered better fitness of the model (Kirrolia et al. 2014). Interestingly, compared with the initial medium with no interactive effects, the RSM optimised condition achieved a 32.3%–fold increase of cell growth yield (OD_600nm_) for our present isolate of bacterial study. According to Kaur and Kaur (2013) the cell growth condition of *E. faecium* GR7 improved with interactive optimisation with experimental data compared with the single factor optimisation approach.

The effect of temperature on cell growth showed that *Enterobacter* sp. DSAAI‐4 could grow from 18°C to 52°C, even though suitable growth was at 30°C–37°C. Tolerance of our isolate with different temperature ranges highlights its potential in different environmental conditions for biocatalyst roles. Physiological growth conditions for *Geobacter sulfurreducens* ^PCAT^ was reported with a growth tolerance from 20°C to 40°C (Viulu et al. 2013), which is comparably in agreement with our minimum temperature range growth tolerance. In addition, Szczerba et al. (2020) reported that 27°C–40°C could support the growth of *E. aerogenes* LU2; however, 37°C was the optimal growth temperature. The interactive effect of various pH ranges significantly affects the growth condition of the present study isolate. The critical role of pH in altering catalytic roles and ionic charges in *Enterobacter cloacae* was reported. In addition, recently Othman et al. (2023) conducted studies with the identification of electrogenic bacteria from waste sludge and reported that the optimum pH and temperature for the growth conditions of *E. cloacae* were 7.5°C and 30°C, respectively.

Moreover, the desirability value obtained for the isolate DSAAI‐4 was 1, which suggests a better optimisation of experimental data for each response variable. The perturbation plot (Figure 5b) for the cell growth (OD_600nm_ value) showed a trend of decrease as pH increases and a gradually lower value as temperature and salt concentration increase. Based on these interaction effects, the effect of pH was clearly demonstrated as a decrease in cell growth as it increases. In line with our finding, a neutral to slightly alkaline pH region supports the optimal performance of electrogenic bacteria (Garimella et al. 2024). Furthermore, optimum pH at neutral regions resulting in better cell growth for *Enterobacter* sp. DSAAI‐4 could highlight the formation of a good biofilm that contributes to better metabolic activities. In line with this Nawaz et al. (2020) discussed that a neutral pH region contributes to the best functioning of microbial enzymes and biofilm performance.

This is the first study to report 16S rRNA gene‐based phylogenetic characterisation of DSAAI‐4 along with growth condition optimisation and substrate utilisation of both commercial and cost‐effective agro‐industrial wastes. Previously, Hemdan et al. (2023) reported an abundance of *Enterobacter* species with electrogenic behaviour using 16S rRNA gene analysis from anode biofilm grown using domestic sludge waste. Here, molecular identification based on sequencing using the 16S rRNA‐encoding gene and BLAST search alignment of the sequences obtained against other DNA sequences deposited in the NCBI GenBank database indicated 98.81% shared similarity with *Enterobacter cloacae* VR27 and 96.4% shared similarity with *Enterobacter huaxiensis*. The genus *Enterobacter* from the phylogenetic tree showed a similarity with 81% bootstrap cutoff at 0.5 nucleotide substitution rates from the nearby evolutionary lineages. In agreement with our findings, Lemoine and Gascuel (2024) showed that branches with bootstrap values ≥ 70% show better identity with a high probability of accurate clustering. Furthermore, strain representation below the 50% cutoff with *Citrobacter*, *Klebsiella* and *Leclercia* (Figure 7) could suggest that our identified isolate is clearly a distinct genus.

## 5 Conclusion

In this study, we optimised experimentally for electrogenic bacteria that can exhibit electrochemical activity in addition to secreting amylase, cellulase, and protease enzymes. Understanding how native electrogenic isolates from environmental sources grow best is crucial to achieving viable cell development for their effective metabolic roles in the production of electric current and the biodegradation of pollutants. The isolate shows improved growth when utilising agro‐industrial waste, BB, and commercial carbon source glucose as a sole carbon source. The analysis of response surface methodology‐based design of optimisation for culture condition studies allows us to better understand the interactive effects between factors and the impact on the responses. This study provides valuable insights, and the eco‐friendly approach of promising EBIs with their physiological adaptations and growth conditions at different temperature ranges, pH, and salt concentration could contribute to serving as a candidate biocatalyst. Utilising untapped agro‐industrial waste as a source of nutrients to support the growth of electrogenic bacteria was proved by our investigation. Our study showed that we can isolate electrogenic bacteria from previously unexplored real industrial wastewater. This was achieved by using an MFC reactor system and a novel bioprospecting approach that utilises 16S rRNA sequencing from the biofilm formed on the anode. In addition, using agro‐industrial waste as a source of nutritional carbon to support the growth of electrogenic bacteria for use in the MFC process is a novel technique. Additionally, the current isolate underwent its first RSM‐based optimisation, which resulted in a 32.3% improvement in cell growth above unoptimised circumstances. Therefore, the newly presented technique has been determined to be a cost‐effective and selective tool for enhancing the growth of electrogenic bacteria. With its broad substrate utilisation potential and adaptable growing conditions, *Enterobacter* sp. DSAAI‐4, an electrogenic species currently isolated from the harsh environment wastewater treatment plant (UASB) reactor, could serve as a biocatalyst in MFC. Its ability to tolerate harsh conditions could make it suitable for MFC applications, as it can adapt to a range of potentially difficult environments within an MFC. Although metabolically diverse activity has been demonstrated by *Enterobacter* sp. DSAAI‐4, more research is required to screen for putative catabolic genes that produce enzymes that effectively metabolise a variety of substrates and to determine how biofilm‐based enzymes contribute to the prevention of electrode biofouling. Electrogenic bacteria and their culture condition, being a promising area, need further investigation on their genetic compositions and metabolite profiles to fully comprehend their increased potential for performance enhancement in MFC.

## Author Contributions

**Getachew Bantihun** and **Andualem Mekonnen:** conceptualisation and methodology. **Getachew Bantihun:** data curation, investigation, original draft preparation, reviewing, editing, formal analysis, software and visualisation. **Andualem Mekonnen** and **Seid Mohammed:** methodology, supervision, validation, project administration, review and editing. **Venkata Kotakadi:** resource, review and editing. All authors have read and approved the final manuscript.

## Conflicts of Interest

The authors declare no conflicts of interest.

## Supporting information


**Data S1:** emi470232‐sup‐0001‐Supinfo.docx.

## Data Availability

Data that support the findings are included in the manuscript. For further information, additional requests can be directed to the corresponding author.
