# Supplementary material for: A Novel Agro‐Waste Formulated Medium Enhanced the Growth of Electrogenic Enterobacter Species Isolated Using Microbial Fuel Cell System: Response Surface Methodology Approach
Source: Environ Microbiol Rep. 2025 Nov 7;17(6):e70232. doi: 10.1111/1758-2229.70232 (PMC12595255; doi:10.1111/1758-2229.70232)
Supplement: Supplementary file 1 — Data S1: emi470232‐sup‐0001‐Supinfo.docx. [file EMI4-17-e70232-s001.docx]

Supplementary file


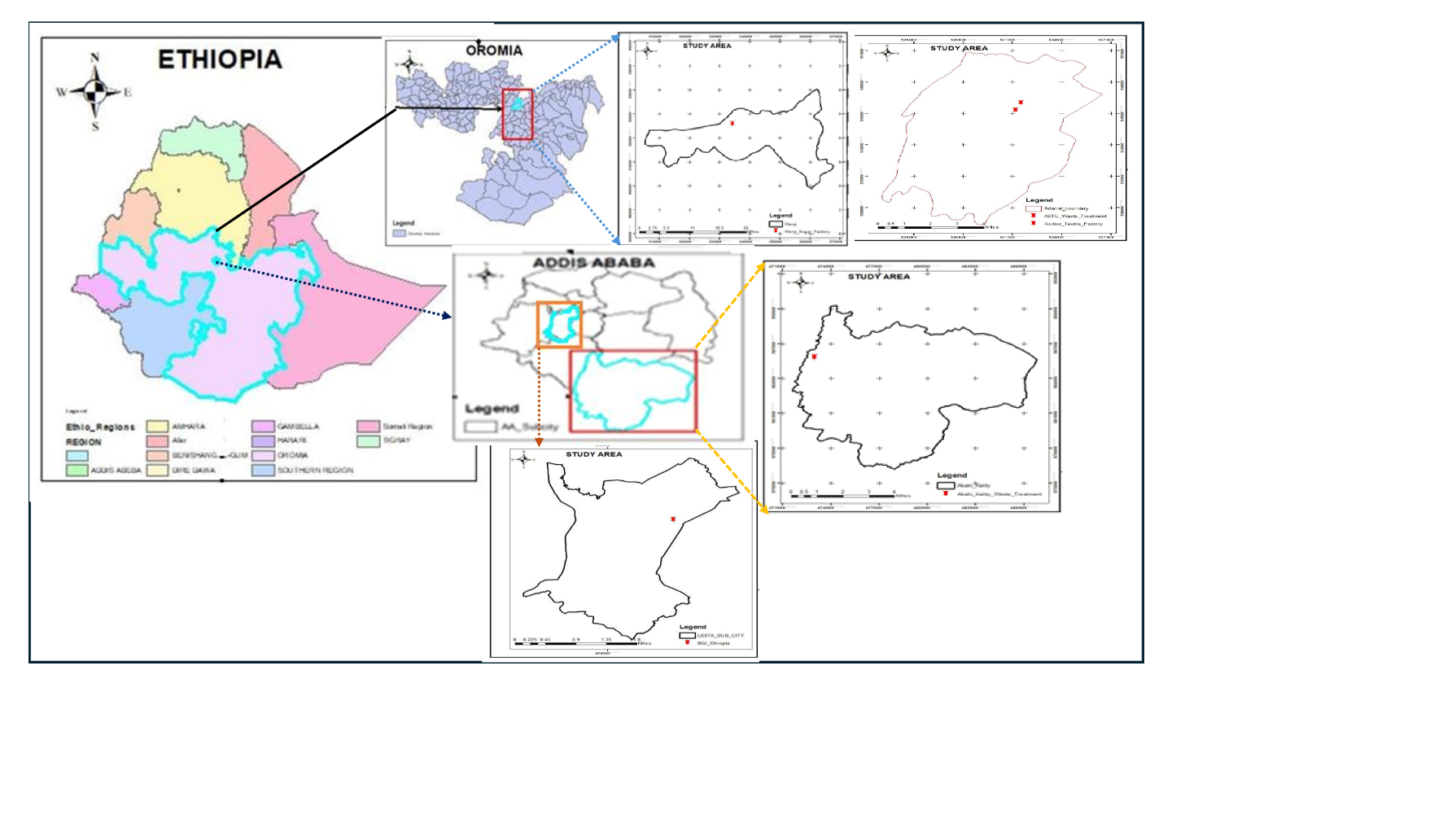

 **Figure A_1_**. Study area map


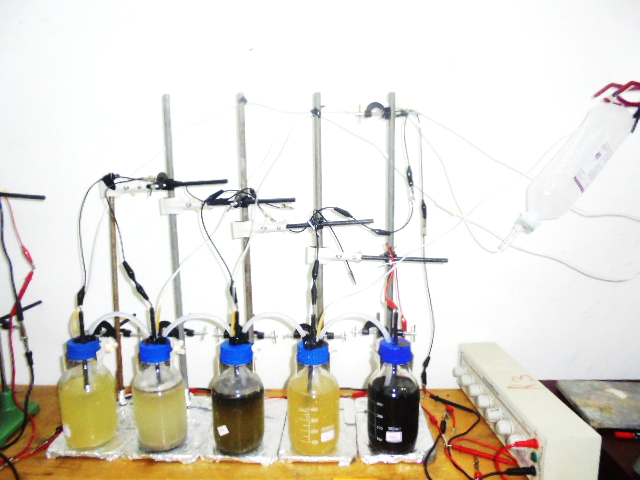


Resistor box

Salt bridge

Gas collector bag

Copper wires

Anodes

Cathode

PGE electrode

**Figure A_2_**. Double chambered MFC set up. MFC was designed using a 500 mL total capacity Schott Duran glass bottle for each and assembled as anodic and cathodic chambers. Salt bridge was used to connect both chambers. Pencil rod graphite electrodes (PGE) were inserted into each camber and connected with copper wires. A resistor box was used to complete voltage monitoring, and a digital multimeter was used to measure the voltages. (Photograph taken by author)


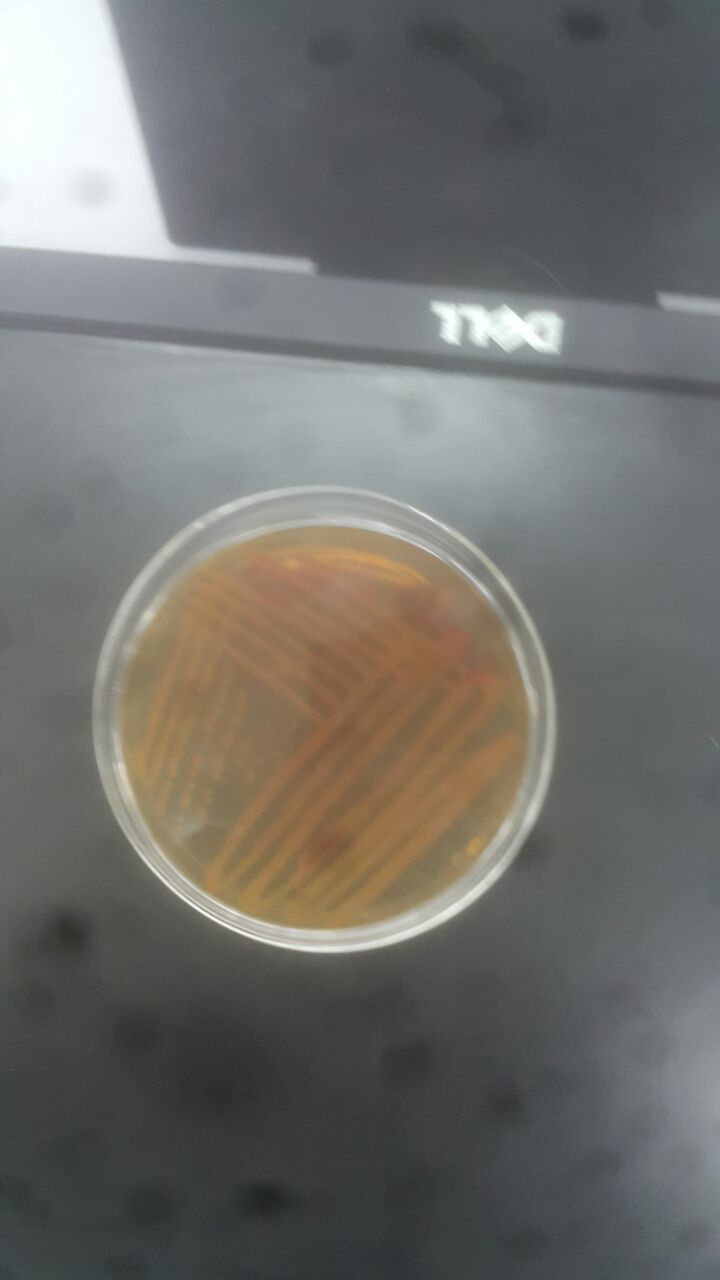


**TXAI-10**


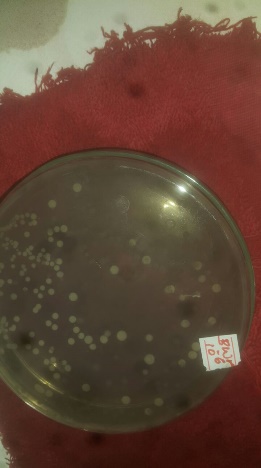

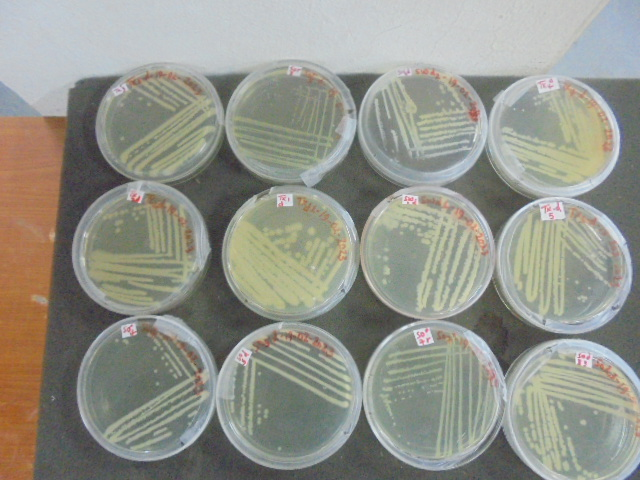

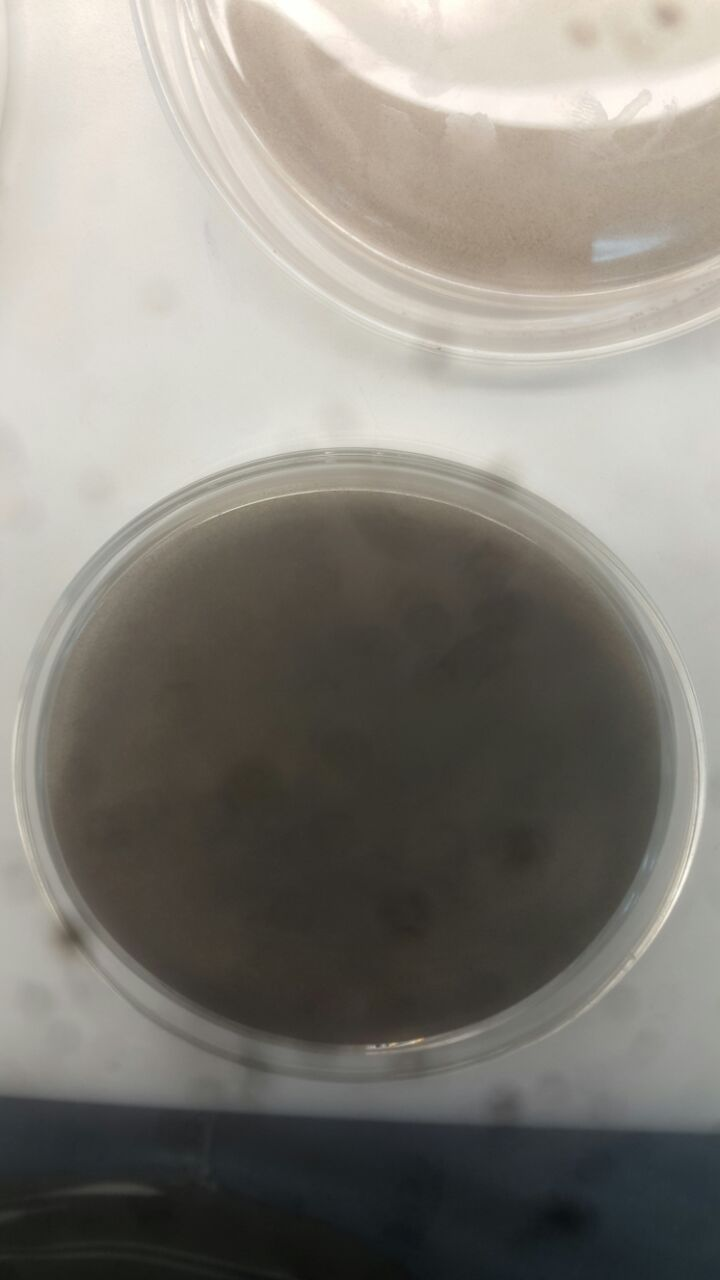

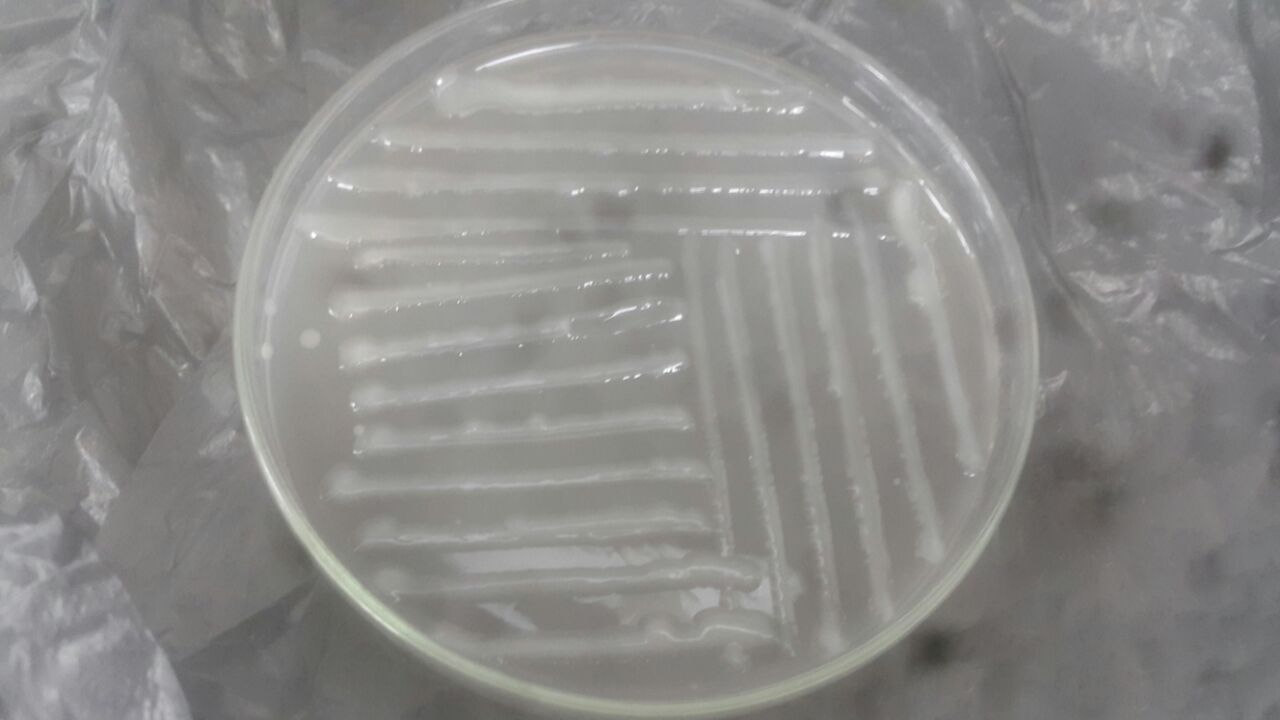

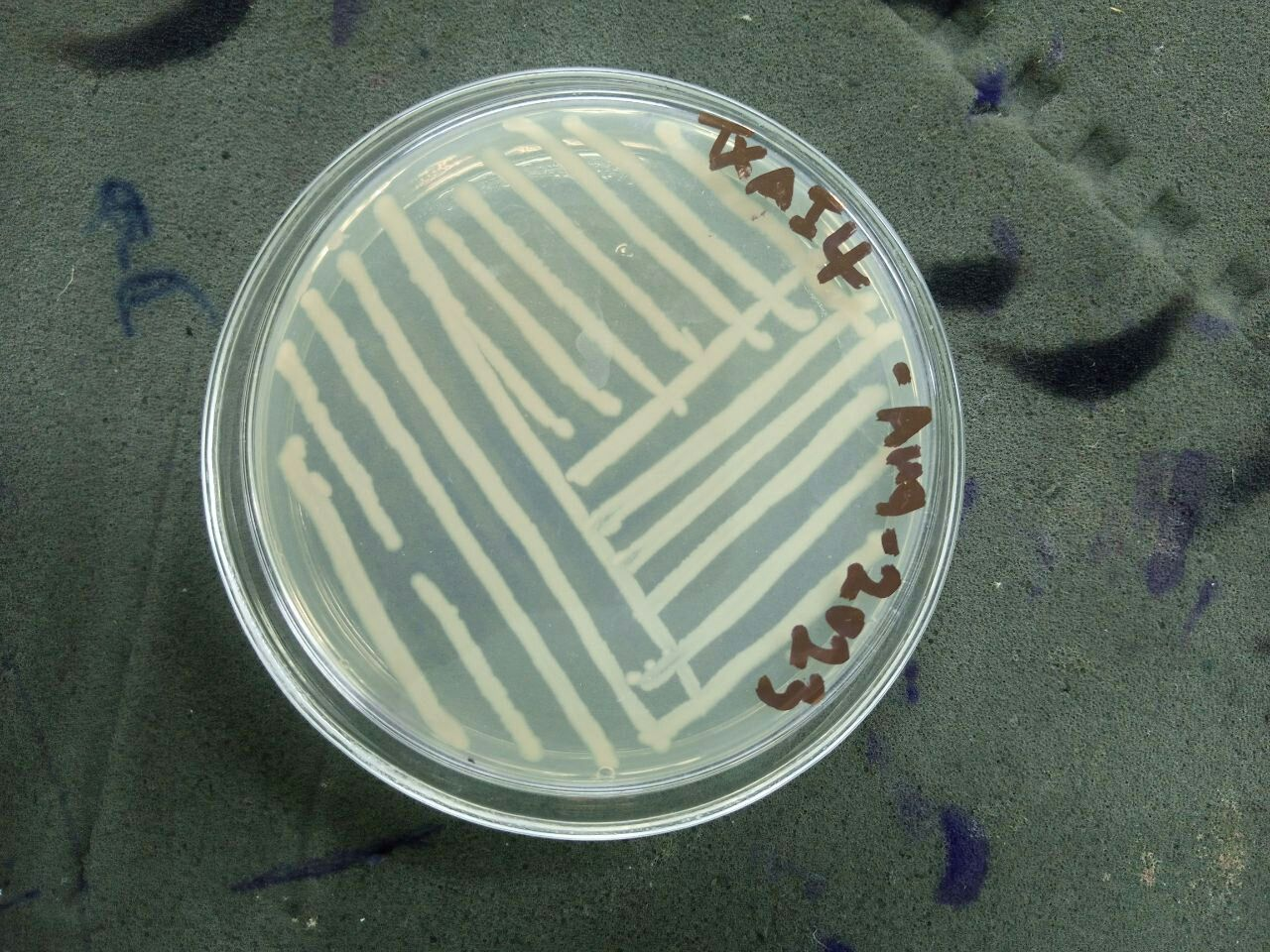


**DSAAI-4**

**SSWI-9**

**MCM medium**

**a**

**b)**

**c)**

**d)**

**e)**

**f)**

**Figure A_3_**. Pure cultures isolates of electrogenic bacteria; **a)** shows Minimal chromogenic media in the presence of electron acceptor MnO_2_ for plate-based primary detection of EB positive isolates; **b)** isolation was obtained from anode biofilm developed from soil sample containing anode of MFCs; **c & f)** isolate from anode biofilm of textile wastewater; **d)** isolate obtained from anode electrode of brewery wastewater. **e)** isolate from anode biofilm of domestic waste sludge.


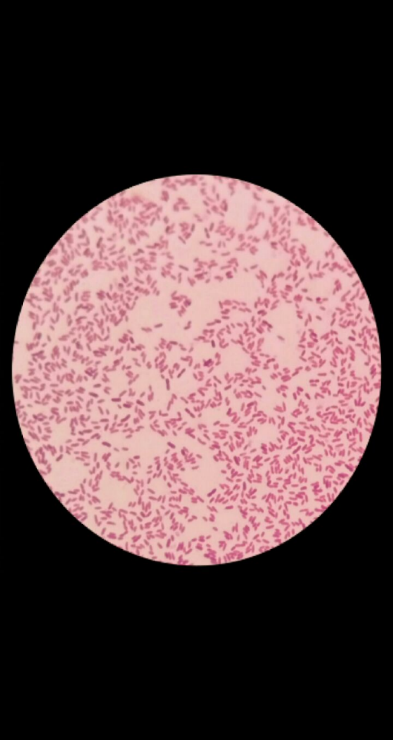

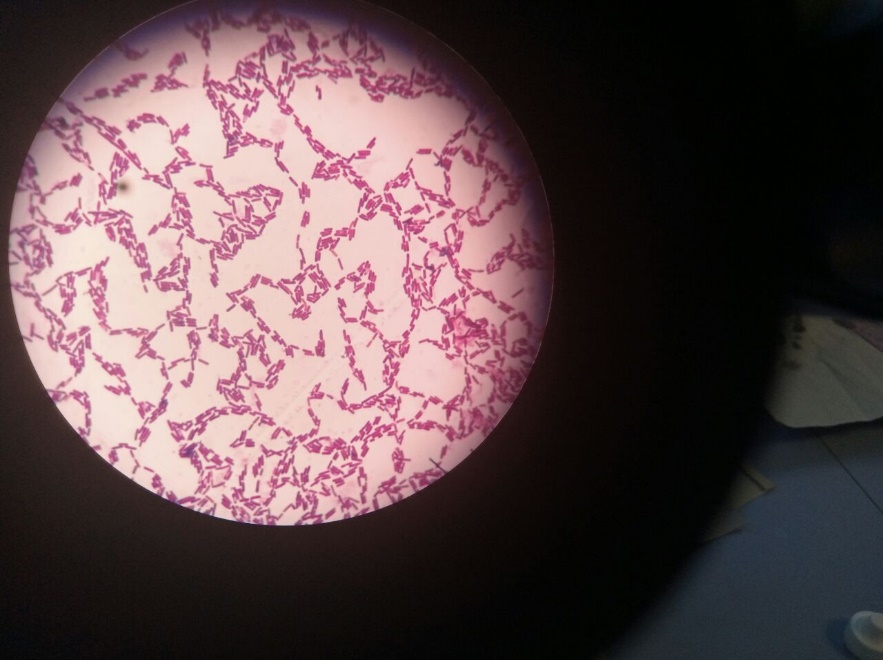

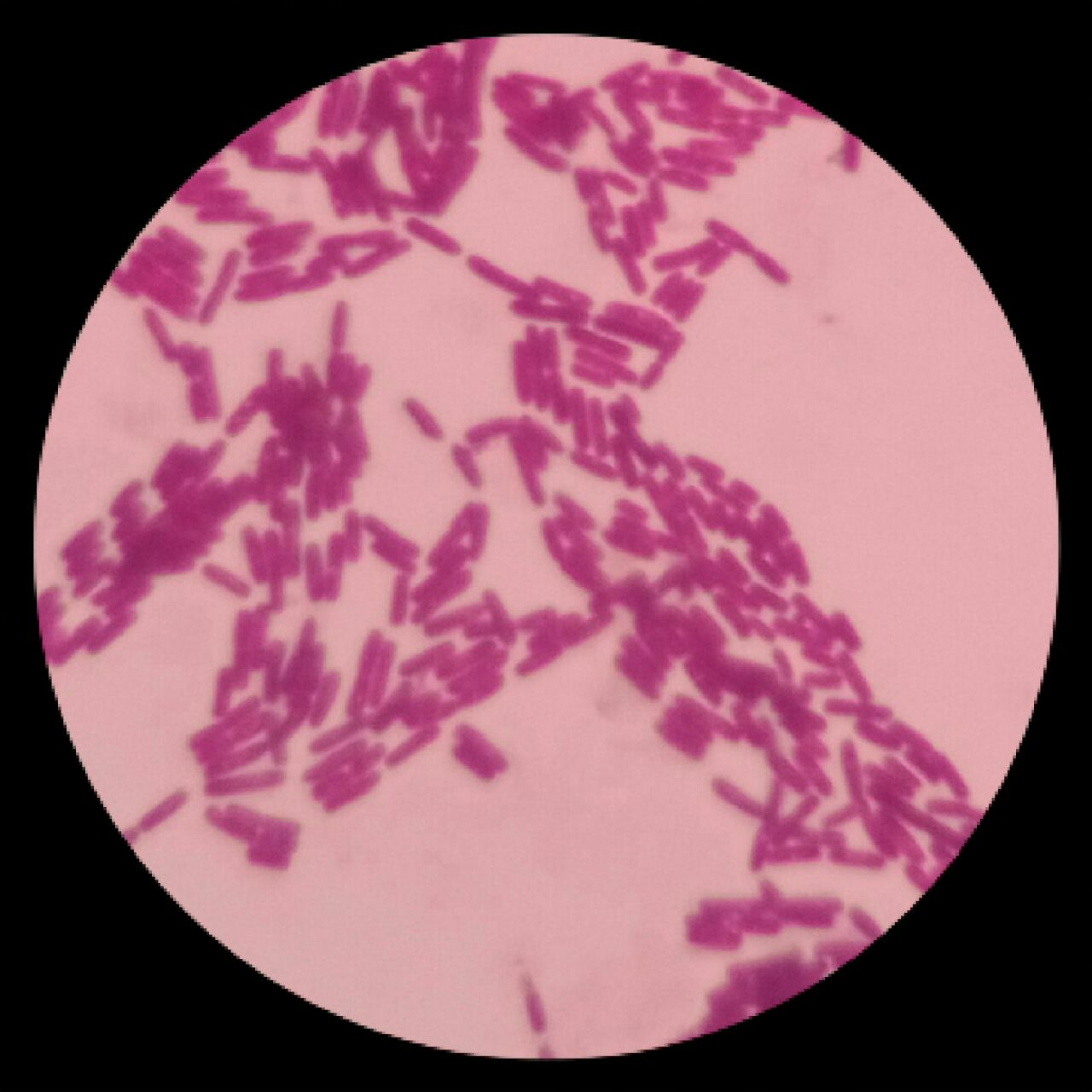

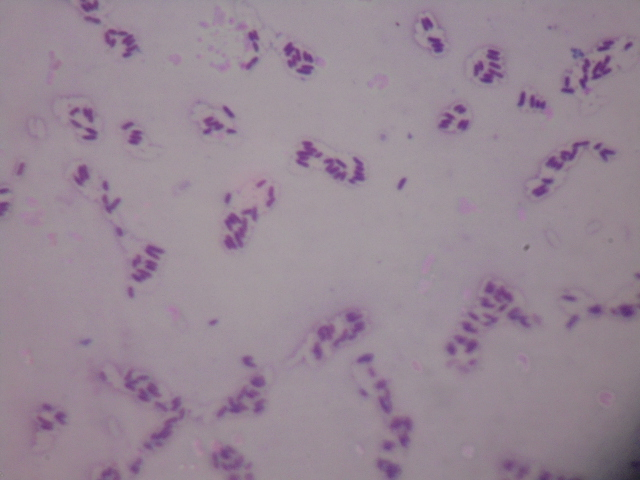


**c)**

**a)**

**d)**


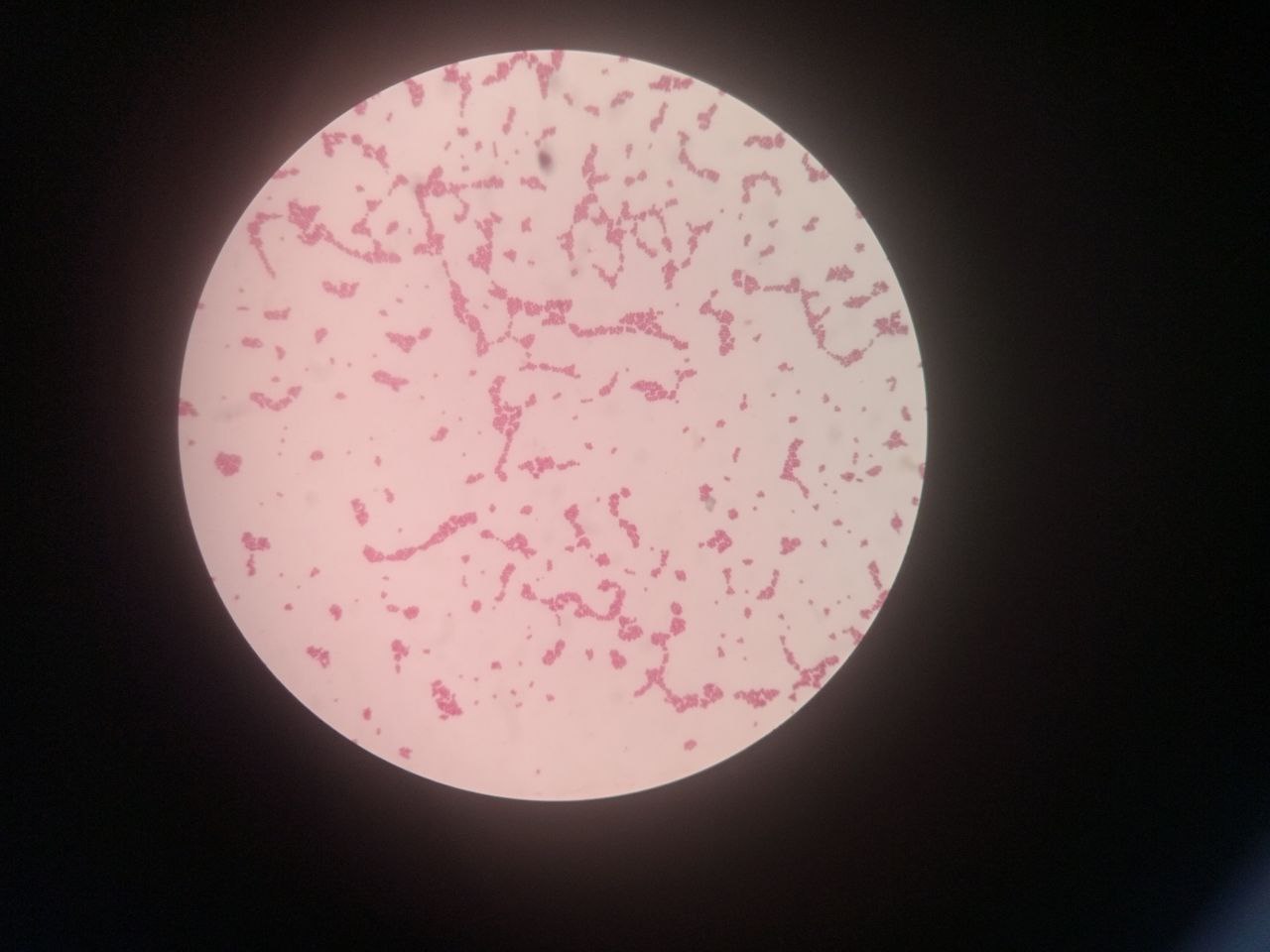


**b)**

**e)**


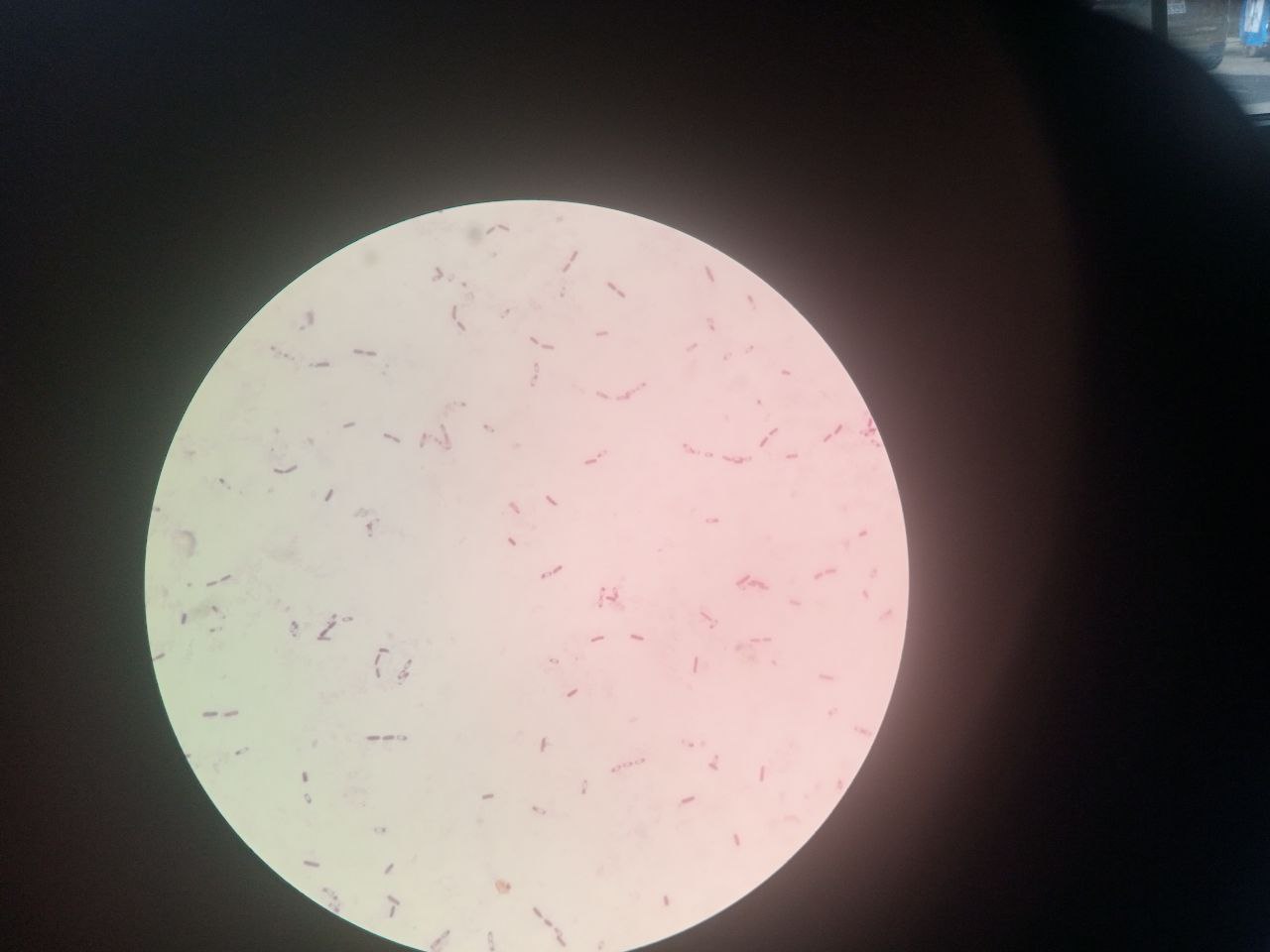


**f)**

**Figure A_4_**. An overview of microscopic Gram stain results of electrogenic bacteria isolates. Figure. **a & b)** DSAAI-4 and DSAAI-5, EBIS of gram staining isolated from anode biofilm developed from domestic waste sludge; **c)**. shows capsulated gram stain for BSAAI-3, isolated from anode biofilm developed from brewery waste sludge; **d) & e)**, shows gram stain for isolates of BWAAI-1 & BWAAI-5, isolated from brewery waste effluent; **f)**, shows short rod bacilli with bipolar staining for EB, BSAAI-3. Images were taken at high power magnification (100x oil immersion objective) observations.

**b)**

**c)**

**d)**

**e)**

**f)**

**a)**


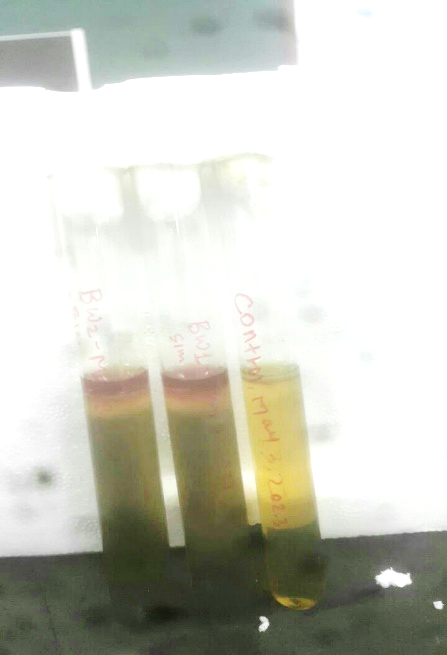

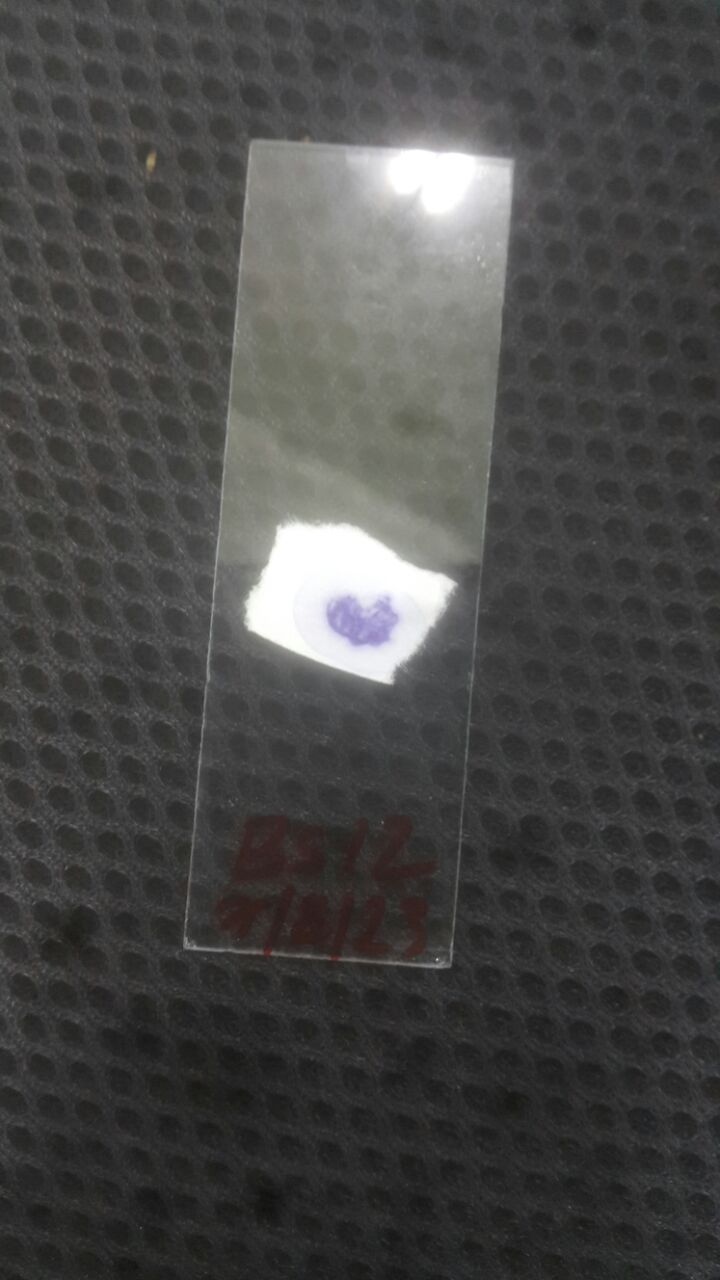

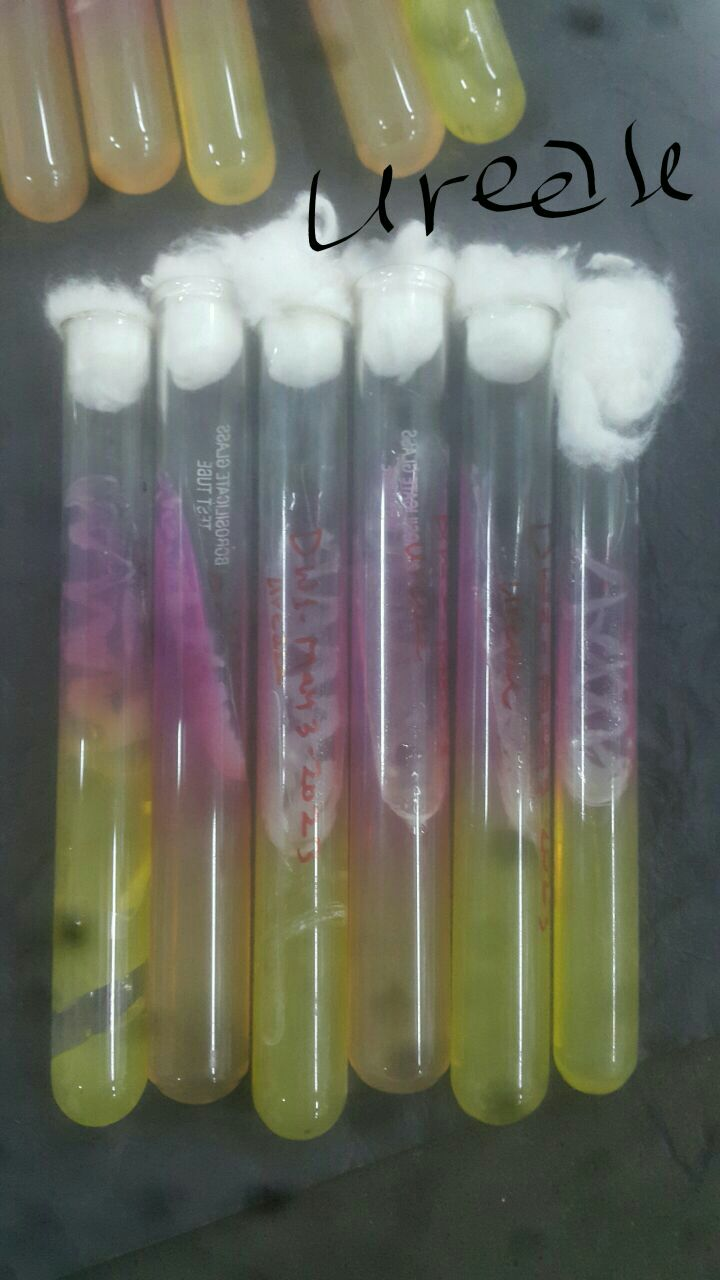

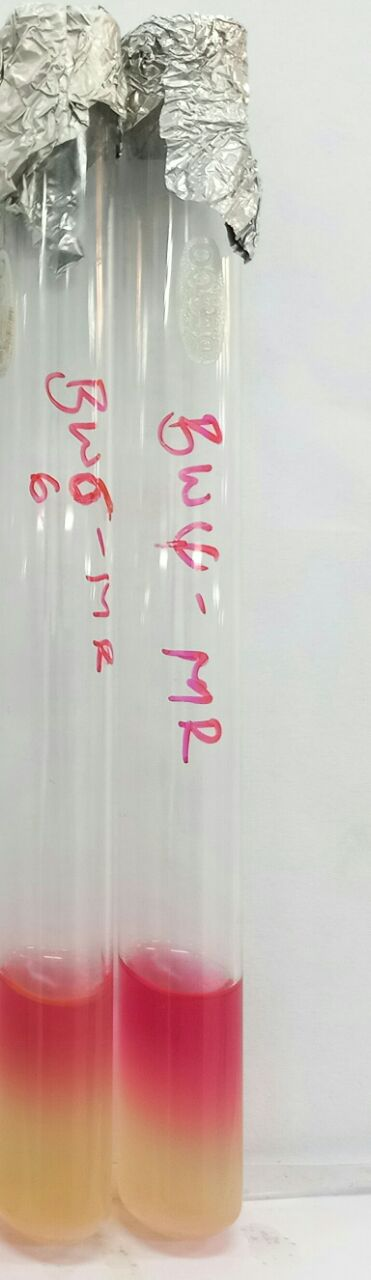

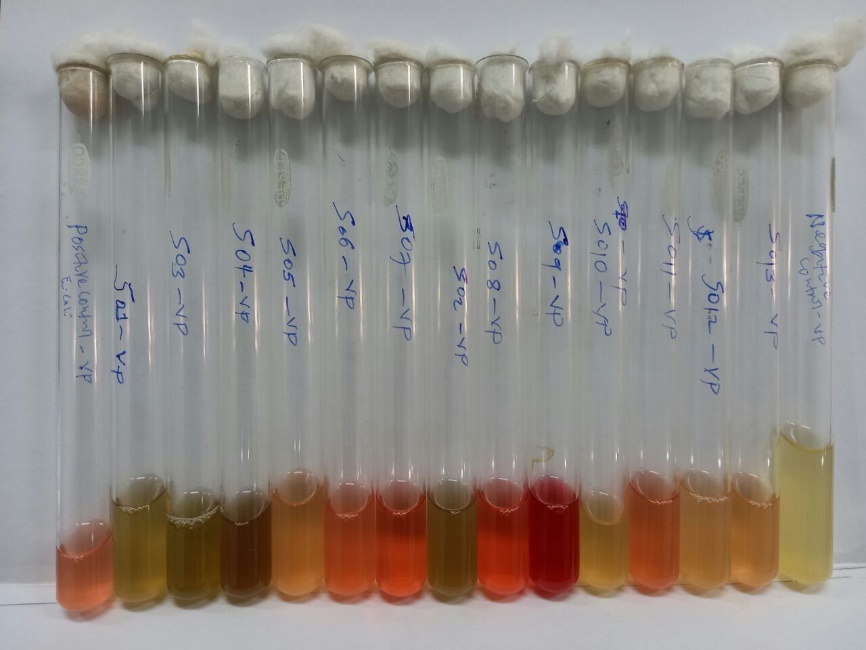

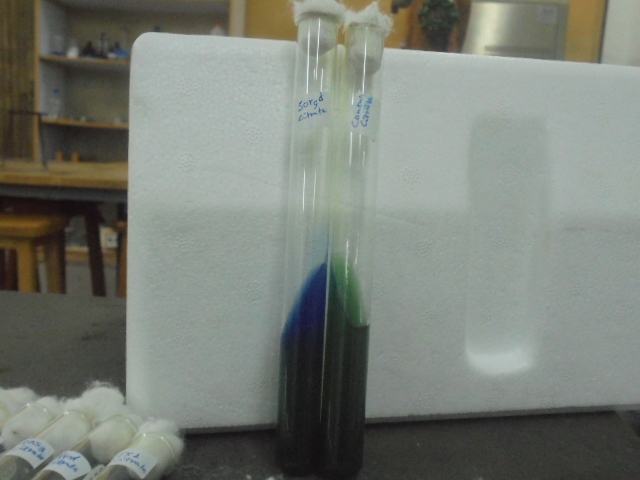


**Figure A_5_**. Images shown for some representative isolates of EB on biochemical test: **a)** positive results for DSAAI-4 showing indole test after drops of Kovac’s reagent **b)** oxidase test, that assays for the presence of cytochrome oxidase **c)** urease hydrolysis test positive results **d)** methyl red test **e)** Voges Proskauer test **f)** citrate utilization test.


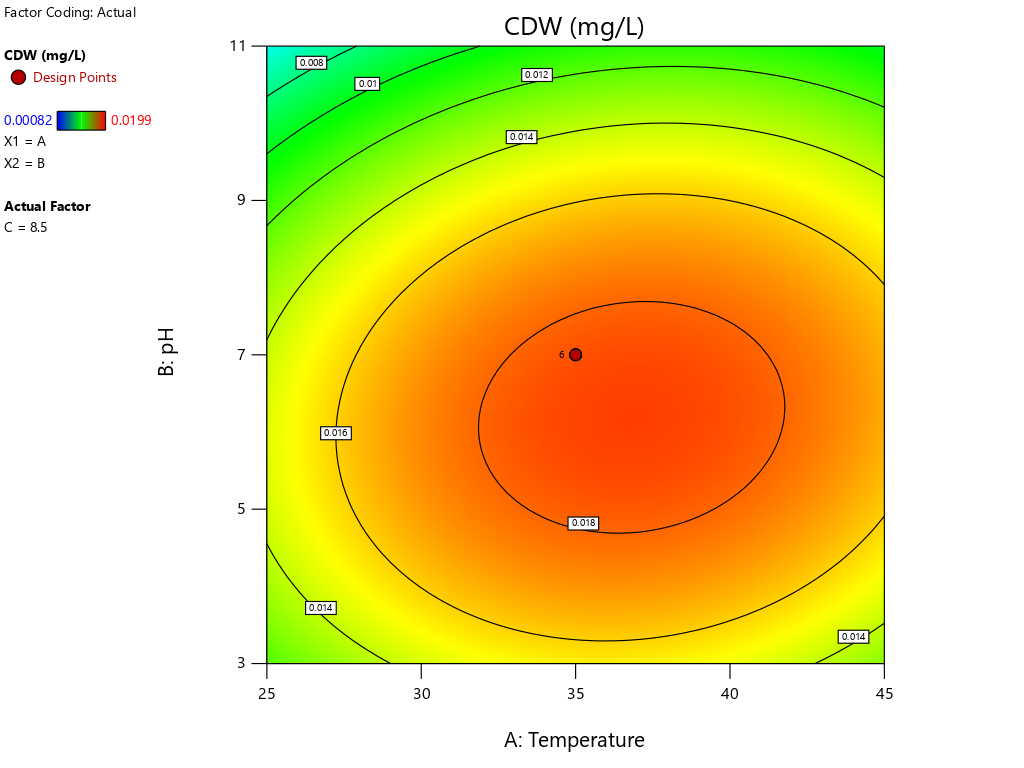

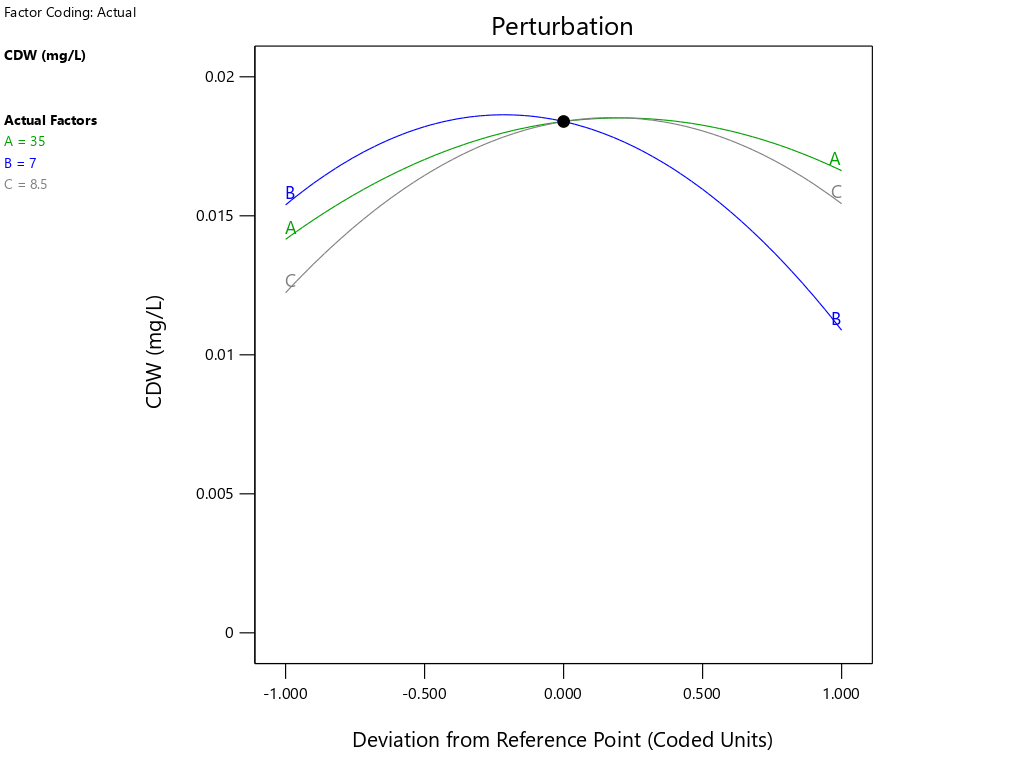

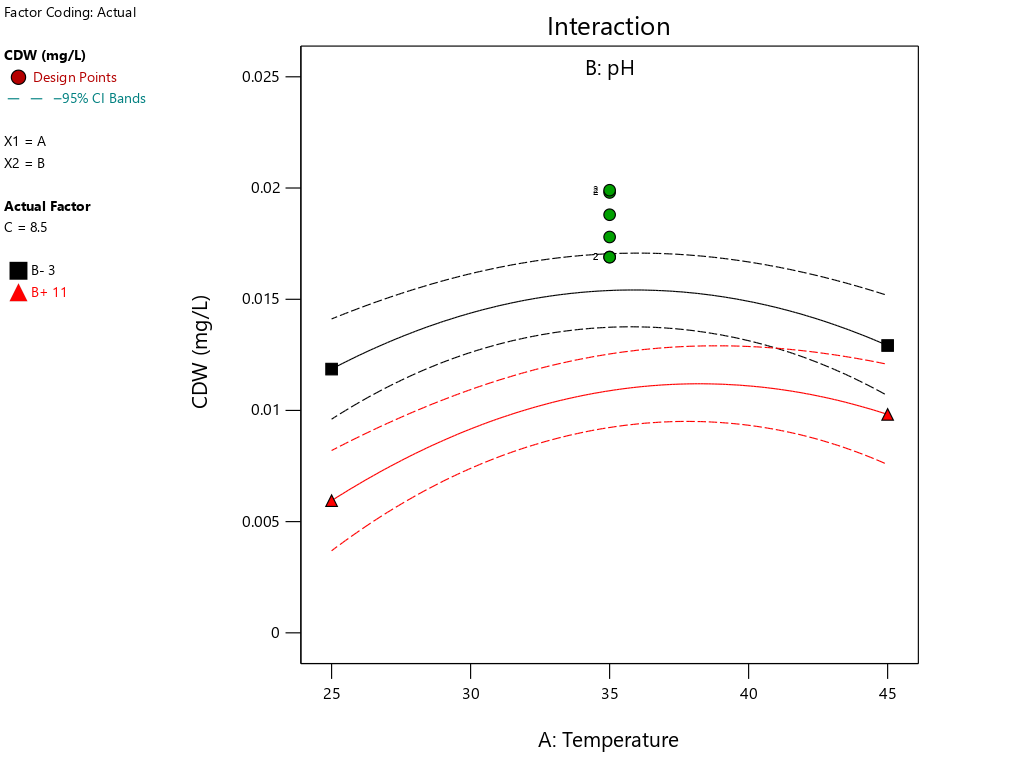

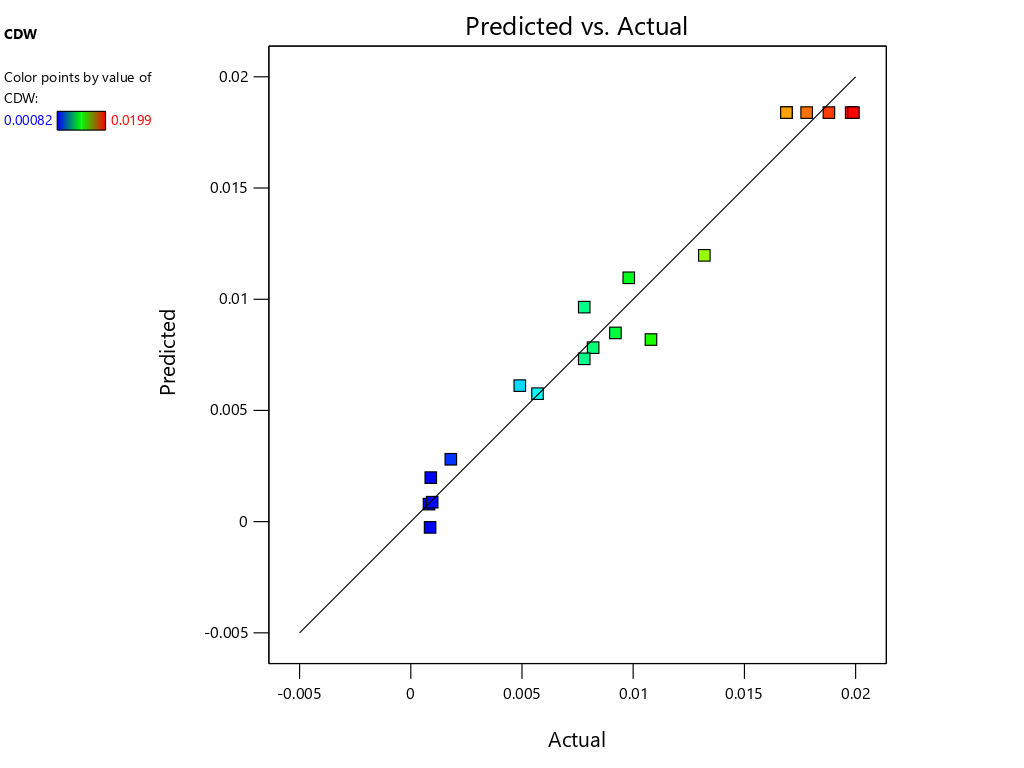

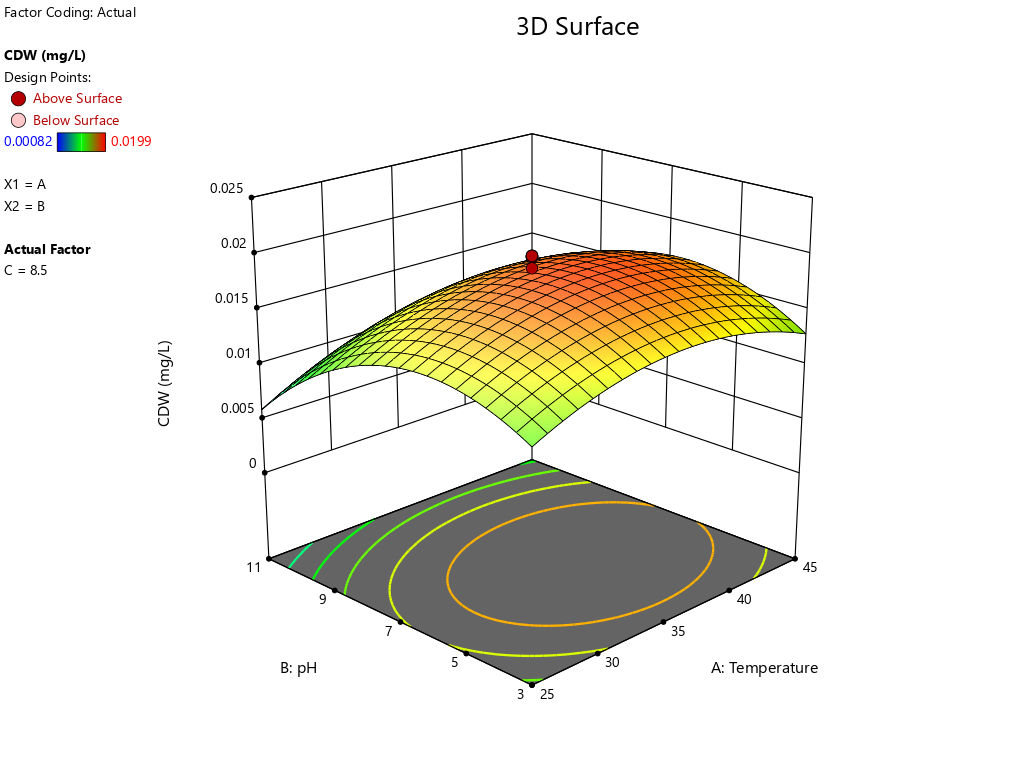

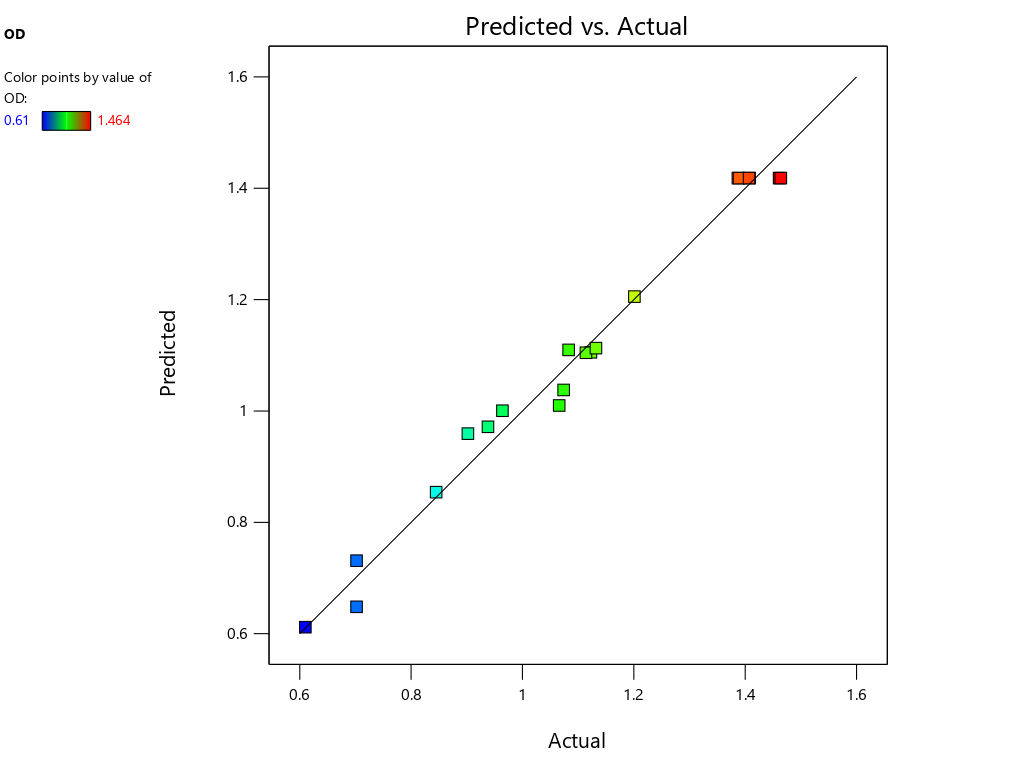


**(a)**

**(b)**

**(c)**

**(d)**

**(e)**

**(f)**

**Figure A_6_**. Response surface plots for OD*_600nm_* and cell dry weight (g/L) for an isolate DSAAI-4: **(a)** perturbation graph representing comparison of influence of temperature (A), pH (B) and salt concentration (C) interactive effects on culture condition in terms of CDW (g/L) to understand interactions, where, as pH increase after the center points influenced CDW value to decrease. **(b)** 2D counter graphs response plots that show interactions among factors on cell dry wight. **(c)** plot showing comparative plots between predicted and actual CDW (g/L) yield. **(d)** interactive effects on CDW response to better understand the best optimum cultivation conditions. **(e)** 3-D response surface plots showing the effect of setting parameters on CDW (g/L) response; accordingly, the graph shows an increase of cell biomass as the pH increases until the neutral region along with the optimum temperature. **(f)** Comparison between predicted and actual CDW(g/L) yield from DSAAI-4 in Central Composite Design.

**Table** **A_1_**. Location details and sample size used for the isolation of electrogenic bacteria

| No. Sampling site | | Sample type | | Sample size | |  |
| --- | --- | --- | --- | --- | --- | --- |
| 1 | BGI Ethiopia brewery factory, Addis Ababa | | Brewery wastewater | | 1 L | |
| 2 | BGI Ethiopia brewery factory, Addis Ababa | | Brewery Activated sludge, from UASB | |  | |
| 3 | Municipal wastewater treatment plant, Addis Ababa | | Activated sludge from   UASB | | 1 kg | |
| 4 | Adama Science and Technology University | | Wastewater | | 1 L | |
| 5 | Wonji Shoa Sugar Factory, Ethiopia | | Sugar factory wastewater | | 1 L | |
| 6 | Adama | | Textile wastewater | | 1 L | |
| 7 | Addis Ababa municipal wastewater landfill site | | Soil sample | | 1 kg | |

Up- flow anaerobic sludge blanket (UASB)

**Table A_2_**_._ **The media and their chemical composition which employed against the recent EBIs**

| S.N. | Type of medium | Their chemical composition |
| --- | --- | --- |
| 1 | Nutrient agar (NA) (g/L), | Peptone- 5, meat extract 1, yeast extract 2, NaCl- 5 and agar 15 per liter of dH_2_O |
| 2 | Minimal chromogenic media (MCM) (g/L) | (Glucose, 2; NH_4_Cl, 0.46; yeast extract, 0.1; peptone, 0.5; K_2_HPO_4_, 5; KH_2_PO_4_, 2.5; MnO_2_,1.5; cysteine, 0.025 and agar,15 |
| 3 | Brain heart infusion agar (BHIA) (g/L) | Brain extract 7.8, dextrose 2, disodium phosphate, 2.5, beef heart extract 9.7, peptone 10, sodium chloride 5, agar 15 and Congo red 0.8 per liter of dH_2_O |
| 4 | CMC agar (g/L) | Carboxymethyl cellulose 26, Ammonium dihydrogen phosphate 1, potassium chloride 1, magnesium sulphate heptahydrate 1, yeast extract 1, agar 15 per liter of dH_2_O |
| 5 | Minimal salt media (MSM) (g/L), | K_2_HPO_4_- 1.73, KH_2_PO_4_ - 0.68, MgSO_4_.7H_2_O- 0.1, NaCl_4_, FeSO_4_.7H_2_O- 0.03, NH_4_NO3- 1, CaCl.2H_2_O- 0.02, C_6_H_12_O_6_ 5 and agar 15 per liter of dH_2_O |
| 6 | Skimmed milk agar (g/L) | skim milk powder 28, tryptone 5, yeast extract dextrose 1 and agar 15 per liter of dH_2_O |
| 7 | Trypticase soy broth (TSB) (g/L), | casein peptone 17, soya peptone 3, NaCl 5, dipotassium hydrogen phosphate 2.5, glucose 2.5 per liter of dH_2_O |

**Table A_3_**. Colony morphological characteristics and microscopic studies of electrogenic isolates

| Isolate Code | Cell shape | Color | Elevation | Surface | Texture | Microscopic observation |
| --- | --- | --- | --- | --- | --- | --- |
| DSAAI-4 | Round | White | Flat | Smooth | Mucoid | Road |
| BSAAI-2 | Irregular | White | Flat | Rough | Mucoid | Cocci |
| BSAAI-7 | Round | Yellow | Flat | Smooth | Viscid | Rod |
| BSAAI-8 | Round | White | Raised | Rough | Mucoid | Rod-bipolar |
| BSAAI-12 | Round | Yellow | Raised | Rough | Mucoid | Rod |
| BSAAI-3 | Irregular | White | Raised | Rough | Viscid | Rod-bipolar |
| BSAAI-16 | Round | White | Raised | Smooth | Mucoid | Rod |
| BSAAI-17 | Round | White | Raised | Smooth | Mucoid | Rod |
| BWAAI-1 | Round | White | Raised | Smooth | Mucoid | Rod |
| BWAAI-2 | Round | White | Flat | Rough | Mucoid | Rod |
| BWAAI-4 | Round | Yellow | Raised | Rough | Viscid | Rod |
| BWAAI-5 | Irregular | White | Flat | Smooth | Viscid | Cocci |
| BWAAI-8 | Irregular | White | Raised | Rough | Mucoid | Rod |
| BWAAI-6 | Round | White | Flat | Smooth | Viscid | Rod |
| DSAAI-1 | Round | Yellow | Flat | Smooth | Mucoid | Rod-capsule |
| DSAAI-5 | Round | White | Flat | Smooth | Viscid | Rod-grape |
| DWASTUI-3 | Round | White | Flat | Smooth | Mucoid | Rod |
| DWASTUI-4 | Irregular | White | Raised | Smooth | Mucoid | Rod |
| DWASTUI-6 | Round | White | Flat | Smooth | Viscid | Rod |
| DWASTUI-7 | Irregular | White | Raised | Smooth | Viscid | Rod |
| SFWWI-7 | Round | White | Raised | Rough | Mucoid | Rod |
| TXAI-3 | Round | White | Flat | Rough | Mucoid | Rod |
| TXAI-4 | Round | White | Raised | Smooth | Viscid | Rod |
| TXAI-10 | Round | Pink | Flat | Smooth | Viscid | Rod |
| SSWI-1 | Round | White | Flat | Smooth | Viscid | Cocci |
| SSWI-9 | Irregular | White | Flat | Smooth | Viscid | Rod |

**Table A_4_**. Triplicates values of optical density (OD) at 570nm for biofilm detection.

| Isolate Code | 96-well plate assay | | | | | Tube method biofilm assay | | | | |
| --- | --- | --- | --- | --- | --- | --- | --- | --- | --- | --- |
|  | Set1 Set2 Set3 Mean Mean±STD | | | | | Set1 Set2 Set3 Mean Mean±STD | | | | |
| DSAAI-4 | 1.572 | 1.419 | 1.202 | 1.4955 | 1.4955±0.18592 | 1.167 | 1.344 | 1.143 | 1.218 | 1.218 ± 0.109777 |
| BSAAI-2 | 1.449 | 1.368 | 1.358 | 1.392 | 1.392±0.049903 | 1.177 | 1.166 | 1.189 | 1.1773 | 1.1773±0.011504 |
| BSAAI-7 | 1.165 | 0.823 | 0.761 | 0.994 | 0.994±0.217571 | 1.355 | 1.433 | 1.455 | 1.414333 | 1.4143±0.0526 |
| BSAAI-8 | 1.591 | 1.448 | 1.367 | 1.468667 | 1.468667±0.113421 | 1.169 | 1.119 | 1.256 | 1.181333 | 1.181333±0.06933 |
| BSAAI1-2 | 1.295 | 1.082 | 1.255 | 1.1885 | 1.1885±0.113209 | 1.171 | 1.092 | 1.154 | 1.139 | 1.139± 0.041581 |
| BSAAI-3 | 1.42 | 1.247 | 1.371 | 1.3335 | 1.3335±0.089168 | 1.077 | 1.044 | 1.025 | 1.048667 | 1.048667±0.026312 |
| BSAAI-16 | 0.845 | 0.848 | 0.671 | 0.8465 | 0.8465±0.101336 | 1.152 | 1.148 | 1.15 | 1.15 | 1.15±0.002 |
| BSAAI-17 | 1.198 | 1.219 | 1.115 | 1.2085 | 1.2085±0.054994 | 1.136 | 1.108 | 1.154 | 1.132667 | 1.132667±0.02318 |
| BWAAI-1 | 1.195 | 1.189 | 1.187 | 1.190333 | 1.190333±0.004163 | 1.225 | 1.08 | 1.134 | 1.146333 | 1.146333±0.073283 |
| BWAAI-2 | 1.166 | 1.079 | 0.987 | 1.077333 | 1.077333±0.089512 | 1.161 | 1.286 | 1.128 | 1.191667 | 1.191667±0.083345 |
| BWAAI-4 | 1.141 | 1.104 | 1.123 | 1.122667 | 1.122667±0.018502 | 1.18 | 1.106 | 1.125 | 1.137 | 1.137±0.038432 |
| BWAAI-5 | 0.987 | 0.881 | 0.828 | 0.898667 | 0.898667±0.080959 | 1.142 | 1.132 | 1.089 | 1.121 | 1.121±0.02816 |
| BWAAI-6 | 0.967 | 0.949 | 0.972 | 0.962667 | 0.962667±0.012097 | 1.136 | 1.127 | 1.083 | 1.115333 | 1.115333±0.028361 |
| BWAAI-8 | 1.001 | 0.913 | 0.904 | 0.939333 | 0.939333±0.053594 | 1.082 | 1.059 | 1.124 | 1.088333 | 1.088333±0.03296 |
| DSAAI-1 | 0.733 | 0.842 | 1.349 | 0.974667 | 0.974667±0.328731 | 1.118 | 1.087 | 1.108 | 1.104333 | 1.104333±0.015822 |
| DSAAI-5 | 1.591 | 1.448 | 1.367 | 1.468667 | 1.468667±0.113421 | 1.232 | 1.213 | 1.156 | 1.200333 | 1.200333±0.039552 |
| DWASTUI-3 | 0.833 | 0.993 | 0.804 | 0.876667 | 0.876667±0.101786 | 0.568 | 0.661 | 0.634 | 0.621 | 0.621±0.047843 |
| DWASTUI-4 | 0.911 | 0.924 | 0.973 | 0.936 | 0.936±0.032696 | 0.929 | 0.921 | 0.876 | 0.908667 | 0.908667±0.028572 |
| DWASTUI-6 | 1.008 | 0.912 | 0.902 | 0.940667 | 0.940667±0.058526 | 0.601 | 0.538 | 0.783 | 0.640667 | 0.640667±0.127226 |
| DWASTUI-7 | 0.959 | 0.989 | 0.943 | 0.963667 | 0.963667±0.023352 | 0.743 | 0.89 | 0.976 | 0.869667 | 0.869667±0.117823 |
| TXWAI-3 | 0.585 | 0.757 | 0.768 | 0.703333 | 0.703333±0.102627 | 0.946 | 0.737 | 0.875 | 0.852667 | 0.852667±0.106275 |
| TXWAI-4 | 0.55 | 0.691 | 0.728 | 0.656333 | 0.656333±0.093927 | 1.163 | 1.108 | 1.145 | 1.138667 | 1.138667±0.028042 |
| TXAI-10 | 0.544 | 0.637 | 0.696 | 0.625667 | 0.625667±0.076631 | 1.146 | 1.074 | 1.121 | 1.113667 | 1.113667±0.036556 |
| SFWWI-7 | 0.877 | 0.595 | 0.764 | 0.75 | 0.75±0.142 | 1.157 | 1.143 | 1.164 | 1.154667 | 1.154667±0.010693 |
| SSWI-1 | 1.122 | 1.275 | 1.135 | 1.177333 | 1.177333±0.084831 | 1.143 | 1.135 | 1.094 | 1.124 | 1.124±0.026287 |
| SSWI-9 | 0.96 | 0.819 | 0.776 | 0.851667 | 0.851667±0.096251 | 1.16 | 0.986 | 1.143 | 1.096333 | 1.096333±0.095929 |

**Table A_5_**. Quadratic model for the experimental run datasets and ANOVA OD response for the selected growth factors

| Source | Sum of  Squares | df | Mean  Square | F-value | p-value |  |
| --- | --- | --- | --- | --- | --- | --- |
| Model | 1.32 | 9 | 0.1462 | 68.00 | < 0.0001 | significant |
| A-Temperature | 0.0285 | 1 | 0.0285 | 13.25 | 0.0045 | significant |
| B-pH | 0.1824 | 1 | 0.1824 | 84.81 | < 0.0001 | significant |
| C-Salt Concentration | 0.0660 | 1 | 0.0660 | 30.68 | 0.0002 | significant |
| AB | 0.0113 | 1 | 0.0113 | 5.27 | 0.0447 | significant |
| AC | 0.0140 | 1 | 0.0140 | 6.52 | 0.0287 | significant |
| BC | 0.0455 | 1 | 0.0455 | 21.13 | 0.0010 | significant |
| A² | 0.2631 | 1 | 0.2631 | 122.32 | < 0.0001 | significant |
| B² | 0.6751 | 1 | 0.6751 | 313.87 | < 0.0001 | significant |
| C² | 0.1958 | 1 | 0.1958 | 91.02 | < 0.0001 | significant |
| Residual | 0.0215 | 10 | 0.0022 |  |  |  |
| Lack of Fit | 0.0155 | 5 | 0.0031 | 2.60 | 0.1590 | not significant |
| Pure Error | 0.0060 | 5 | 0.0012 |  |  |  |
| Cor Total | 1.34 | 19 |  |  |  |  |

*The* ***Model F-value*** *of 68.00 implies the model is significant. There is only a 0.01% chance that an F-value this large could occur due to noise.*

**Table A_6_**. Quadratic model for the experimental datasets and ANOVA for CDW (g/L) response for the selected growth factors

| Source | Sum of Squares | df | Mean Square | F-value | p-value |  |
| --- | --- | --- | --- | --- | --- | --- |
| Model | 0.0009 | 9 | 0.0001 | 34.13 | < 0.0001 | significant |
| A-Temperature | 0.0000 | 1 | 0.0000 | 7.38 | 0.0217 | significant |
| B-pH | 0.0001 | 1 | 0.0001 | 24.62 | 0.0006 | significant |
| C-Salt Concentration | 0.0000 | 1 | 0.0000 | 12.42 | 0.0055 | significant |
| AB | 3.976E-06 | 1 | 3.976E-06 | 1.41 | 0.2622 |  |
| AC | 0.0000 | 1 | 0.0000 | 10.20 | 0.0096 | significant |
| BC | 6.337E-06 | 1 | 6.337E-06 | 2.25 | 0.1645 |  |
| A² | 0.0001 | 1 | 0.0001 | 46.17 | < 0.0001 | significant |
| B² | 0.0004 | 1 | 0.0004 | 141.25 | < 0.0001 | significant |
| C² | 0.0003 | 1 | 0.0003 | 106.37 | < 0.0001 | significant |
| Residual | 0.0000 | 10 | 2.816E-06 |  |  |  |
| Lack of Fit | 0.0000 | 5 | 3.784E-06 | 2.05 | 0.2253 | not significant |
| Pure Error | 9.244E-06 | 5 | 1.849E-06 |  |  |  |
| Cor Total | 0.0009 | 19 |  |  |  |  |

*The* ***Model F-value*** *of 34.13 implies the model is significant. There is only a 0.01% chance that an F-value this large could occur due to noise.*

**Table A_7_**. **Descriptive statistical analysis on effect of commercial carbon sources against culture condition of DSAAI-4**

| Effect of various carbon sources against OD_600nm_ for *Enterobacter* sp. DSAAI-4 growth and their variation | | Mean Difference | Std. Error | Sig. | 95% Confidence Interval | |
| --- | --- | --- | --- | --- | --- | --- |
|  |  |  |  |  | **Lower Bound** | **Upper Bound** |
| Effect of Glucose against OD at 600nm for *Enterobacter* sp. DSAAI-4 growth | Effect of Fructose against OD at 600nm for *Enterobacter* sp. DSAAI-4 growth | .0477500^*^ | .0031075 | .000 | .040584 | .054916 |
|  | Effect of Mannitol against OD at 600nm for *Enterobacter* sp. DSAAI-4 growth | .0225567^*^ | .0031075 | .000 | .015391 | .029723 |
|  | Effect of sucrose against OD at 600nm for *Enterobacter* sp. DSAAI-4 growth | .0938100^*^ | .0031075 | .000 | .086644 | .100976 |
| Effect of Fructose against OD at 600nm for *Enterobacter* sp. DSAAI-4 growth | Effect of Glucose against OD at 600nm for *Enterobacter* sp. DSAAI-4 growth | -.0477500^*^ | .0031075 | .000 | -.054916 | -.040584 |
|  | Effect of Mannitol against OD at 600nm for *Enterobacter* sp. DSAAI-4 growth | -.0251933^*^ | .0031075 | .000 | -.032359 | -.018027 |
|  | Effect of sucrose against OD at 600nm for *Enterobacter* sp. DSAAI-4 growth | .0460600^*^ | .0031075 | .000 | .038894 | .053226 |
| Effect of Mannitol against OD at 600nm for *Enterobacter* sp. DSAAI-4 growth | Effect of Glucose against OD at 600nm for *Enterobacter* sp. DSAAI-4 growth | -.0225567^*^ | .0031075 | .000 | -.029723 | -.015391 |
|  | Effect of Fructose against OD at 600nm for *Enterobacter* sp. DSAAI-4 growth | .0251933^*^ | .0031075 | .000 | .018027 | .032359 |
|  | Effect of sucrose against OD at 600nm for *Enterobacter* sp. DSAAI-4 growth | .0712533^*^ | .0031075 | .000 | .064087 | .078419 |
| Effect of sucrose against OD at 600nm for Enterobacter sp. DSAAI-4 growth | Effect of Glucose against OD at 600nm for *Enterobacter* sp. DSAAI-4 growth | -.0938100^*^ | .0031075 | .000 | -.100976 | -.086644 |
|  | Effect of Fructose against OD at 600nm for *Enterobacter* sp. DSAAI-4 growth | -.0460600^*^ | .0031075 | .000 | -.053226 | -.038894 |
|  | Effect of Mannitol against OD at 600nm for *Enterobacter* sp. DSAAI-4 growth | -.0712533^*^ | .0031075 | .000 | -.078419 | -.064087 |
| *. The mean difference is significant at the 0.05 level. | | | | | | |

**Table A_8_**. **Multiple comparisons test for various carbons sources against *Enterobacter* sp. DSAAI-4 growth in terms of CDW (g/L) at LSD test using One-Way-ANOVA**

| Effect of various carbon sources against CDW (g/L) for *Enterobacter* sp. DSAAI-4 growth and their variation | | Mean Difference | Std. Error | Sig. | 95% Confidence Interval | |
| --- | --- | --- | --- | --- | --- | --- |
|  |  |  |  |  | **Lower Bound** | **Upper Bound** |
| Effect of Glucose against CDW (g/L) for *Enterobacter* sp. DSAAI-4 growth | Effect of Fructose against CDW (g/L) for *Enterobacter* sp. DSAAI-4 growth | 0.0902^*^ | 0.00127 | .001 | .0061 | .0120 |
|  | Effect of Mannitol against CDW (g/L) for *Enterobacter* sp. DSAAI-4 growth | .00328^*^ | 0.00127 | .033 | .003 | .0062 |
|  | Effect of sucrose against CDW (g/L) for *Enterobacter* sp. DSAAI-4 growth | .00728^*^ | 0.00127 | .001 | .0043 | .0102 |
| Effect of Fructose against CDW (g/L) for *Enterobacter* sp. DSAAI-4 growth | Effect of Glucose against CDW (g/L) for *Enterobacter* sp. DSAAI-4 growth | -.00902^*^ | 0.00127 | .001 | -.0120 | -.0061 |
|  | Effect of Mannitol against CDW (g/L) for *Enterobacter* sp. DSAAI-4 growth | -.00574^*^ | 0.00127 | .002 | -.0087 | -.0028 |
|  | Effect of sucrose against CDW (g/L) for *Enterobacter* sp. DSAAI-4 growth | -.00723^*^ | 0.00127 | .021 | -.0047 | .0012 |
| Effect of Mannitol against CDW (g/L) for *Enterobacter* sp. DSAAI-4 growth | Effect of Glucose against CDW (g/L) for *Enterobacter* sp. DSAAI-4 growth | -.00328^*^ | 0.00127 | .033 | -.0062 | -.0003 |
|  | Effect of Fructose against CDW (g/L) for *Enterobacter* sp. DSAAI-4 growth | .00574^*^ | 0.00127 | .002 | .0028 | .0087 |
|  | Effect of sucrose against CDW (g/L) for *Enterobacter* sp. DSAAI-4 growth | .00400^*^ | 0.00127 | .014 | .0011 | .0069 |
| Effect of sucrose against CDW (g/L) for *Enterobacter* sp. DSAAI-4 growth | Effect of Glucose against CDW (g/L) for *Enterobacter* sp. DSAAI-4 growth | -.00728^*^ | 0.00127 | .000 | -.0102 | -.0043 |
|  | Effect of Fructose against CDW (g/L) for *Enterobacter* sp. DSAAI-4 growth | .00174^*^ | 0.00127 | .021 | -.0012 | .0047 |
|  | Effect of Mannitol against CDW (g/L) for *Enterobacter* sp. DSAAI-4 growth | -.00400^*^ | .00127 | .014 | -.0069 | -.0011 |
|  | ***. The mean difference is significant at the 0.05 level.** | | | | | |

**Table A_9_**. **Multiple comparisons test for various nitrogen sources against *Enterobacter* sp. DSAAI-4 growth in terms of OD at 600nm at LSD test using One-Way-ANOVA**

| Effect of various nitrogen sources against OD at 600nm for *Enterobacter* sp. DSAAI-4 growth and their variation | | Mean Difference | Std. Error | Sig. | 95% Confidence Interval | | |
| --- | --- | --- | --- | --- | --- | --- | --- |
|  | |  |  |  | **Lower Bound** | **Upper Bound** | |
| Effect of Yeast extract against OD at 600nm for *Enterobacter* sp. DSAAI-4 growth | Effect of Peptone against OD at 600nm for *Enterobacter* sp. DSAAI-4 growth | .13416^*^ | .01324 | .000 | .1047 | .1637 | |
|  | Effect of Ammonium sulfate against OD at 600nm for *Enterobacter* sp. DSAAI-4 growth | .31327^*^ | .01324 | .000 | .2838 | .3428 | |
|  | Effect of Potassium nitrate against OD at 600nm for *Enterobacter* sp. DSAAI-4 growth | .23740^*^ | .01324 | .000 | .2079 | .2669 | |
|  | Effect of Urea against OD at 600nm for *Enterobacter* sp. DSAAI-4 growth | .22603^*^ | .01324 | .000 | .1965 | .2555 | |
| Effect of Peptone against OD at 600nm for *Enterobacter* sp. DSAAI-4 growth | Effect of Yeast extract against OD at 600nm for *Enterobacter* sp. DSAAI-4 growth | -.13416^*^ | .01324 | .000 | -.1637 | -.1047 | |
|  | Effect of Ammonium sulfate against OD at 600nm for *Enterobacter* sp. DSAAI-4 growth | .17911^*^ | .01324 | .000 | .1496 | .2086 | |
|  | Effect of Potassium nitrate against OD at 600nm for *Enterobacter* sp. DSAAI-4 growth | .10324^*^ | .01324 | .000 | .0737 | .1327 | |
|  | Effect of Urea against OD at 600nm for *Enterobacter* sp. DSAAI-4 growth | .09188^*^ | .01324 | .000 | .0624 | .1214 | |
| Effect of Ammonium sulfate against OD at 600nm for *Enterobacter* sp. DSAAI-4 growth | Effect of Yeast extract against OD at 600nm for *Enterobacter* sp. DSAAI-4 growth | -.31327^*^ | .01324 | .000 | -.3428 | -.2838 | |
|  | Effect of Peptone against OD at 600nm for *Enterobacter* sp. DSAAI-4 growth | -.17911^*^ | .01324 | .000 | -.2086 | -.1496 | |
|  | Effect of Potassium nitrate against OD at 600nm for *Enterobacter* sp. DSAAI-4 growth | -.07587^*^ | .01324 | .700 | -.1054 | .0464 | |
|  | Effect of Urea against OD at 600nm for *Enterobacter* sp. DSAAI-4 growth | -.08723^*^ | .01324 | .051 | -.1167 | .0577 | |
| Effect of Potassium nitrate OD at 600nm for Enterobacter sp. DSAAI-4 growth | Effect of Yeast extract against OD at 600nm for *Enterobacter* sp. DSAAI-4 growth | -.23740^*^ | .01324 | .000 | -.2669 | -.2079 | |
|  | Effect of Peptone against OD at 600nm for *Enterobacter* sp. DSAAI-4 growth | -.10324^*^ | .01324 | .000 | -.1327 | -.0737 |  |
|  | Effect of Ammonium sulfate against OD at 600nm for *Enterobacter* sp. DSAAI-4 growth | -.07587^*^ | .01324 | .700 | -.0464 | .1054 |  |
|  | Effect of Urea against OD at 600nm for *Enterobacter* sp. DSAAI-4 growth | -.01137 | .01324 | .411 | -.0409 | .0181 |  |
| Effect of Urea against OD at 600nm for Enterobacter sp. DSAAI-4 growth | Effect of Yeast extract against OD at 600nm for *Enterobacter* sp. DSAAI-4 growth | -.22603^*^ | .01324 | .000 | -.2555 | -.1965 |  |
|  | Effect of Peptone against OD at 600nm for *Enterobacter* sp. DSAAI-4 growth | -.09188^*^ | .01324 | .000 | -.1214 | -.0624 |  |
|  | Effect of Ammonium sulfate against OD at 600nm for *Enterobacter* sp. DSAAI-4 growth | .08723^*^ | .01324 | 0.51 | .0577 | .1167 |  |
|  | Effect of Potassium nitrate against OD at 600nm for *Enterobacter* sp. DSAAI-4 growth | .01137 | .01324 | .411 | -.0181 | .0409 |  |
| *. The mean difference is significant at the 0.05 level. | |  |  |  |  |  |  |

**Table A_10_**. **Multiple comparisons test for various nitrogen sources against *Enterobacter* sp. DSAAI-4 growth in terms of CDW (g/L) at LSD test using One-Way-ANOVA**

| Effect of various nitrogen sources against CDW (g/L) for *Enterobacter* sp. DSAAI-4 growth and their variation | | Mean Difference | Std. Error | Sig. | 95% Confidence Interval | | |
| --- | --- | --- | --- | --- | --- | --- | --- |
|  | |  |  |  | **Lower Bound** | **Upper Bound** | |
| Effect of Yeast extract against CDW (g/L) for *Enterobacter* sp. DSAAI-4 growth | Effect of Peptone against CDW (g/L) for *Enterobacter* sp. DSAAI-4 growth | .00205^*^ | .00031 | .000 | .1047 | .0027 | |
|  | Effect of Ammonium sulfate against CDW (g/L) for *Enterobacter* sp. DSAAI-4 growth | .01088^*^ | .00031 | .000 | .0102 | .0116 | |
|  | Effect of Potassium nitrate against CDW (g/L) for *Enterobacter* sp. DSAAI-4 growth | .00588^*^ | .00031 | .000 | .0052 | .0066 | |
|  | Effect of Urea against CDW (g/L) for *Enterobacter* sp. DSAAI-4 growth | .00480^*^ | .00031 | .000 | -.0041 | .0055 | |
| Effect of Peptone against CDW (g/L) for *Enterobacter* sp. DSAAI-4 growth | Effect of Yeast extract against CDW (g/L) for *Enterobacter* sp. DSAAI-4 growth | -.00205^*^ | .00031 | .000 | -.0027 | -.0014 | |
|  | Effect of Ammonium sulfate against CDW (g/L) for *Enterobacter* sp. DSAAI-4 growth | .00883^*^ | .00031 | .000 | .0081 | .0095 | |
|  | Effect of Potassium nitrate against CDW (g/L) for *Enterobacter* sp. DSAAI-4 growth | .00383^*^ | .00031 | .000 | .0031 | .0045 | |
|  | Effect of Urea against CDW (g/L) for *Enterobacter* sp. DSAAI-4 growth | .00275^*^ | .00031 | .000 | .0021 | .0034 | |
| Effect of Ammonium sulfate against CDW (g/L) for *Enterobacter* sp. DSAAI-4 growth | Effect of Yeast extract against CDW (g/L) for *Enterobacter* sp. DSAAI-4 growth | -.01088^*^ | .00031 | .000 | -.0116 | -.0102 | |
|  | Effect of Peptone against CDW (g/L) for *Enterobacter* sp. DSAAI-4 growth | -.00883^*^ | .00031 | .000 | -.0095 | -.0081 | |
|  | Effect of Potassium nitrate against CDW (g/L) for *Enterobacter* sp. DSAAI-4 growth | -.00500^*^ | .00031 | .000 | -.0057 | -.0043 | |
|  | Effect of Urea against CDW (g/L) for *Enterobacter* sp. DSAAI-4 growth | -.00608^*^ | .00031 | .000 | -.0068 | -.0054 | |
| Effect of Potassium nitrate CDW (g/L) for Enterobacter sp. DSAAI-4 growth | Effect of Yeast extract against CDW (g/L) for *Enterobacter* sp. DSAAI-4 growth | -.00588^*^ | .00031 | .000 | -.0066 | -.0052 | |
|  | Effect of Peptone against CDW (g/L) for *Enterobacter* sp. DSAAI-4 growth | -.00383^*^ | .00031 | .000 | -.0045 | -.0031 |  |
|  | Effect of Ammonium sulfate against CDW (g/L) for *Enterobacter* sp. DSAAI-4 growth | -.00500^*^ | .00031 | .000 | -.0043 | .0057 |  |
|  | Effect of Urea against CDW (g/L) for *Enterobacter* sp. DSAAI-4 growth | -.00108^*^ | .00031 | .005 | -.0018 | -.0004 |  |
| Effect of Urea against CDW (g/L) for Enterobacter sp. DSAAI-4 growth | Effect of Yeast extract against CDW (g/L) for *Enterobacter* sp. DSAAI-4 growth | -.00480^*^ | .00031 | .000 | -.0055 | -.0041 |  |
|  | Effect of Peptone against CDW (g/L) for *Enterobacter* sp. DSAAI-4 growth | -.00275^*^ | .00031 | .000 | -.0034 | -.0021 |  |
|  | Effect of Ammonium sulfate against CDW (g/L) for *Enterobacter* sp. DSAAI-4 growth | .00608^*^ | .00031 | 0.00 | .0054 | .0068 |  |
|  | Effect of potassium nitrate against CDW (g/L) for *Enterobacter* sp. DSAAI-4 growth | .00108^*^ | .00031 | .005 | .0004 | .0018 |  |
| *. The mean difference is significant at the 0.05 level. | |  |  |  |  |  |  |

**Table A_11_**. **Multiple comparisons test for various agro - industrial waste as a cost-effective carbon sources against *Enterobacter* sp. DSAAI-4 growth in terms of OD at 600nm at LSD test using One-Way-ANOVA**

| Effect of various nitrogen sources against OD at 600nm for *Enterobacter* sp. DSAAI-4 growth and their variation | | Mean Difference | Std. Error | Sig. | 95% Confidence Interval | | |
| --- | --- | --- | --- | --- | --- | --- | --- |
|  | |  |  |  | **Lower Bound** | **Upper Bound** | |
| Effect of Teff straw against OD at 600nm for *Enterobacter* sp. DSAAI-4 growth | Effect of Bagasse against OD at 600nm for *Enterobacter* sp. DSAAI-4 growth | -.33530^*^ | .03101 | .000 | -.4018 | -.2688 | |
|  | Effect of Molasses against OD at 600nm for *Enterobacter* sp. DSAAI-4 growth | -.36130^*^ | .03101 | .000 | -.4278 | -.2948 | |
|  | Effect of Barley bran against OD at 600nm for *Enterobacter* sp. DSAAI-4 growth | -.52600^*^ | .03101 | .000 | -.5925 | -.4595 | |
|  | Effect of Wheat bran against OD at 600nm for *Enterobacter* sp. DSAAI-4 growth | -.05800 | .03101 | .082 | -.1245 | .0085 | |
|  | Effect of Cotton waste against OD at 600nm for *Enterobacter* sp. DSAAI-4 growth | .08050^*^ | .03101 | .021 | .0140 | .1470 | |
|  | Effect of Activated sludge against OD at 600nm for *Enterobacter* sp. DSAAI-4 growth | -.37747^*^ | .03101 | .000 | -.4440 | -.3110 | |
| Effect of Bagasse against OD at 600nm for *Enterobacter* sp. DSAAI-4 growth | Effect of Teff straw against OD at 600nm for *Enterobacter* sp. DSAAI-4 growth | .33530^*^ | .03101 | .000 | .2688 | .4018 | |
|  | Effect of Molasses against OD at 600nm for *Enterobacter* sp. DSAAI-4 growth | -.02600 | .03101 | .416 | -.0925 | .0405 | |
|  | Effect of Barley bran against OD at 600nm for *Enterobacter* sp. DSAAI-4 growth | -.19070^*^ | .03101 | .000 | -.2572 | -.1242 | |
|  | Effect of Wheat bran against OD at 600nm for *Enterobacter* sp. DSAAI-4 growth | .27730^*^ | .03101 | .000 | .2108 | .3438 | |
|  | Effect of Cotton waste against OD at 600nm for *Enterobacter* sp. DSAAI-4 growth | .41580^*^ | .03101 | .000 | .3493 | .4823 | |
|  | Effect of Activated sludge against OD at 600nm for *Enterobacter* sp. DSAAI-4 growth | -.04217 | .03101 | .195 | -.1087 | .0243 | |
| Effect of Molasses against OD at 600nm for *Enterobacter* sp. DSAAI-4 growth | Effect of Teff straw against OD at 600nm for *Enterobacter* sp. DSAAI-4 growth | .36130^*^ | .03101 | .000 | .2948 | .4278 | |
|  | Effect of Bagasse against OD at 600nm for *Enterobacter* sp. DSAAI-4 growth | .02600 | .03101 | .416 | -.0405 | .0925 | |
|  | Effect of Barley bran against OD at 600nm for *Enterobacter* sp. DSAAI-4 growth | -.16470^*^ | .03101 | .000 | -.2312 | -.0982 | |
|  | Effect of Wheat bran against OD at 600nm for *Enterobacter* sp. DSAAI-4 growth | .30330^*^ | .03101 | .000 | .2368 | .3698 | |
|  | Effect of Cotton waste against OD at 600nm for *Enterobacter* sp. DSAAI-4 growth | .44180^*^ | .03101 | .000 | .3753 | .5083 | |
|  | Effect of Activated sludge against OD at 600nm for *Enterobacter* sp. DSAAI-4 growth | -.01617 | .03101 | .610 | -.0827 | .0503 | |
| Effect of Barley bran OD at 600nm for Enterobacter sp. DSAAI-4 growth | Effect of Teff straw against OD at 600nm for *Enterobacter* sp. DSAAI-4 growth | .52600^*^ | .03101 | .000 | .4595 | .5925 | |
|  | Effect of Bagasse against OD at 600nm for *Enterobacter* sp. DSAAI-4 growth | .19070^*^ | .03101 | .000 | .1242 | .2572 |  |
|  | Effect of Molasses against OD at 600nm for *Enterobacter* sp. DSAAI-4 growth | .16470^*^ | .03101 | .000 | .0982 | .2312 |  |
|  | Effect of Wheat bran against OD at 600nm for *Enterobacter* sp. DSAAI-4 growth | .46800^*^ | .03101 | .000 | .4015 | .5345 |  |
|  | Effect of Cotton waste against OD at 600nm for *Enterobacter* sp. DSAAI-4 growth | .60650^*^ | .03101 | .000 | .5400 | .6730 |  |
|  | Effect of Activated sludge against OD at 600nm for *Enterobacter* sp. DSAAI-4 growth | .14853^*^ | .03101 | .000 | .0820 | .2150 |  |
| Effect of Wheat bran against OD at 600nm for Enterobacter sp. DSAAI-4 growth | Effect of Teff straw against OD at 600nm for *Enterobacter* sp. DSAAI-4 growth | .05800 | .03101 | .082 | -.0085 | .1245 |  |
|  | Effect of Bagasse against OD at 600nm for *Enterobacter* sp. DSAAI-4 growth | -.27730^*^ | .03101 | .000 | -.3438 | -.2108 |  |
|  | Effect of Molasses against OD at 600nm for *Enterobacter* sp. DSAAI-4 growth | -.30330^*^ | .03101 | .000 | -.3698 | -.2368 |  |
|  | Effect of Barley bran against OD at 600nm for *Enterobacter* sp. DSAAI-4 growth | -.46800^*^ | .03101 | .000 | -.5345 | -.4015 |  |
|  | Effect of Cotton waste against OD at 600nm for *Enterobacter* sp. DSAAI-4 growth | .13850^*^ | .03101 | .001 | .0720 | .2050 |  |
|  | Effect of Activated sludge against OD at 600nm for *Enterobacter* sp. DSAAI-4 growth | -.31947^*^ | .03101 | .000 | -.3860 | -.2530 |  |
| Effect of Cotton waste against OD at 600nm for Enterobacter sp. DSAAI-4 growth | Effect of Teff straw against OD at 600nm for *Enterobacter* sp. DSAAI-4 growth | -.08050^*^ | .03101 | .021 | -.1470 | -.0140 |  |
|  | Effect of Bagasse against OD at 600nm for *Enterobacter* sp. DSAAI-4 growth | -.41580^*^ | .03101 | .000 | -.4823 | -.3493 |  |
|  | Effect of Molasses against OD at 600nm for *Enterobacter* sp. DSAAI-4 growth | -.44180^*^ | .03101 | .000 | -.5083 | -.3753 |  |
|  | Effect of Barley bran against OD at 600nm for *Enterobacter* sp. DSAAI-4 growth | -.60650^*^ | .03101 | .000 | -.6730 | -.5400 |  |
|  | Effect of Wheat bran against OD at 600nm for Enterobacter sp. DSAAI-4 growth | -.13850^*^ | .03101 | .001 | -.2050 | -.0720 |  |
|  | Effect of Activated sludge against OD at 600nm for *Enterobacter* sp. DSAAI-4 growth | -.45797^*^ | .03101 | .000 | -.5245 | -.3915 |  |
| Effect of Activated sludge against OD at 600nm for *Enterobacter* sp. DSAAI-4 growth | Effect of Teff straw against OD at 600nm for *Enterobacter* sp. DSAAI-4 growth | .37747^*^ | .03101 | .000 | .3110 | .4440 |  |
|  | Effect of Bagasse against OD at 600nm for *Enterobacter* sp. DSAAI-4 growth | .04217 | .03101 | .195 | -.0243 | .1087 |  |
|  | Effect of Molasses against OD at 600nm for *Enterobacter* sp. DSAAI-4 growth | .01617 | .03101 | .610 | -.0503 | .0827 |  |
|  | Effect of Barley bran against OD at 600nm for *Enterobacter* sp. DSAAI-4 growth | -.14853^*^ | .03101 | .000 | -.2150 | -.0820 |  |
|  | Effect of Wheat bran against OD at 600nm for Enterobacter sp. DSAAI-4 growth | .31947^*^ | .03101 | .000 | .2530 | .3860 |  |
|  | Effect of Cotton waste against OD at 600nm for Enterobacter sp. DSAAI-4 growth | .45797^*^ | .03101 | .000 | .3915 | .5245 |  |
| *. The mean difference is significant at the 0.05 level. | |  |  |  |  |  |  |

**Table A_12_**. **Multiple comparisons test for various agro - industrial waste as a cost-effective carbon sources against *Enterobacter* sp. DSAAI-4 growth in terms of CDW (g/L) at LSD test using One-Way-ANOVA**

| Effect of various nitrogen sources against CDW (g/L) for *Enterobacter* sp. DSAAI-4 growth and their variation | | Mean Difference | Std. Error | Sig. | 95% Confidence Interval | | |
| --- | --- | --- | --- | --- | --- | --- | --- |
|  | |  |  |  | **Lower Bound** | **Upper Bound** | |
| Effect of Teff straw against CDW (g/L) for *Enterobacter* sp. DSAAI-4 growth | Effect of Bagasse against CDW (g/L) for *Enterobacter* sp. DSAAI-4 growth | -.00572^*^ | .00055 | .000 | -.0069 | -.0045 | |
|  | Effect of Molasses against CDW (g/L) for *Enterobacter* sp. DSAAI-4 growth | -.00642^*^ | .00055 | .000 | -.0076 | -.0052 | |
|  | Effect of Barley bran against CDW (g/L) for *Enterobacter* sp. DSAAI-4 growth | -.00792^*^ | .00055 | .000 | -.0091 | -.0067 | |
|  | Effect of Wheat bran against CDW (g/L) for *Enterobacter* sp. DSAAI-4 growth | .00031 | .00055 | .037 | -.0009 | .0015 | |
|  | Effect of Cotton waste against CDW (g/L) for *Enterobacter* sp. DSAAI-4 growth | .00099 | .00055 | .014 | -.0002 | .0022 | |
|  | Effect of Activated sludge against CDW (g/L) for *Enterobacter* sp. DSAAI-4 growth | -.00700^*^ | .00055 | .000 | -.0082 | -.0058 | |
| Effect of Bagasse against CDW (g/L) for *Enterobacter* sp. DSAAI-4 growth | Effect of Teff straw against CDW (g/L) for *Enterobacter* sp. DSAAI-4 growth | .00572^*^ | .00055 | .000 | .0045 | .0069 | |
|  | Effect of Molasses against CDW (g/L) for *Enterobacter* sp. DSAAI-4 growth | -.00070 | .00055 | .0223 | -.0019 | .0005 | |
|  | Effect of Barley bran against CDW (g/L) for *Enterobacter* sp. DSAAI-4 growth | -.00220^*^ | .00055 | .001 | -.0034 | -.0010 | |
|  | Effect of Wheat bran against CDW (g/L) for *Enterobacter* sp. DSAAI-4 growth | .00602^*^ | .00055 | .000 | .0048 | .0072 | |
|  | Effect of Cotton waste against CDW (g/L) for *Enterobacter* sp. DSAAI-4 growth | .00671^*^ | .00055 | .000 | .0055 | .0079 | |
|  | Effect of Activated sludge against CDW (g/L) for *Enterobacter* sp. DSAAI-4 growth | -.00128^*^ | .00055 | .036 | -.0025 | -.0001 | |
| Effect of Molasses against CDW (g/L) for *Enterobacter* sp. DSAAI-4 growth | Effect of Teff straw against CDW (g/L) for *Enterobacter* sp. DSAAI-4 growth | .00642^*^ | .00055 | .000 | .0052 | .0076 | |
|  | Effect of Bagasse against CDW (g/L) for *Enterobacter* sp. DSAAI-4 growth | .00070 | .00055 | .0223 | -.0005 | .0019 | |
|  | Effect of Barley bran against CDW (g/L) for *Enterobacter* sp. DSAAI-4 growth | -.00150^*^ | .00055 | .017 | -.0027 | -.0003 | |
|  | Effect of Wheat bran against CDW (g/L) for *Enterobacter* sp. DSAAI-4 growth | .00673^*^ | .00055 | .000 | .0055 | .0079 | |
|  | Effect of Cotton waste against CDW (g/L) for *Enterobacter* sp. DSAAI-4 growth | .00741^*^ | .00055 | .000 | .0062 | .0086 | |
|  | Effect of Activated sludge against CDW (g/L) for *Enterobacter* sp. DSAAI-4 growth | -.00058 | .00055 | .313 | -.0018 | .0006 | |
| Effect of Barley bran CDW (g/L) for Enterobacter sp. DSAAI-4 growth | Effect of Teff straw against CDW (g/L) for *Enterobacter* sp. DSAAI-4 growth | .00792^*^ | .00055 | .000 | .0067 | .0091 | |
|  | Effect of Bagasse against CDW (g/L) for *Enterobacter* sp. DSAAI-4 growth | .00220^*^ | .00055 | .001 | .0010 | .0034 |  |
|  | Effect of Molasses against CDW (g/L) for *Enterobacter* sp. DSAAI-4 growth | .00150^*^ | .00055 | .017 | .0003 | .0027 |  |
|  | Effect of Wheat bran against CDW (g/L) for *Enterobacter* sp. DSAAI-4 growth | .00823^*^ | .00055 | .000 | .0070 | .0094 |  |
|  | Effect of Cotton waste against CDW (g/L) for *Enterobacter* sp. DSAAI-4 growth | .00891^*^ | .00055 | .000 | .0077 | .0101 |  |
|  | Effect of Activated sludge against CDW (g/L) for *Enterobacter* sp. DSAAI-4 growth | .00092 | .00055 | .016 | -.0003 | .0021 |  |
| Effect of Wheat bran against CDW (g/L) for Enterobacter sp. DSAAI-4 growth | Effect of Teff straw against CDW (g/L) for *Enterobacter* sp. DSAAI-4 growth | -.00031 | .00055 | .037 | -.0015 | .0009 |  |
|  | Effect of Bagasse against CDW (g/L) for *Enterobacter* sp. DSAAI-4 growth | -.00602^*^ | .00055 | .000 | -.0072 | -.0048 |  |
|  | Effect of Molasses against CDW (g/L) for *Enterobacter* sp. DSAAI-4 growth | -.00673^*^ | .00055 | .000 | -.0079 | -.0055 |  |
|  | Effect of Barley bran against CDW (g/L) for *Enterobacter* sp. DSAAI-4 growth | -.00823^*^ | .00055 | .000 | -.0094 | -.0070 |  |
|  | Effect of Cotton waste against CDW (g/L) for *Enterobacter* sp. DSAAI-4 growth | .00068 | .00055 | .236 | -.0005 | .0019 |  |
|  | Effect of Activated sludge against CDW (g/L) for *Enterobacter* sp. DSAAI-4 growth | -.00730^*^ | .00055 | .000 | -.0085 | -.0061 |  |
| Effect of Cotton waste against CDW (g/L) for Enterobacter sp. DSAAI-4 growth | Effect of Teff straw against CDW (g/L) for *Enterobacter* sp. DSAAI-4 growth | -.00099 | .00055 | .014 | -.0022 | .0002 |  |
|  | Effect of Bagasse against CDW (g/L) for *Enterobacter* sp. DSAAI-4 growth | -.00671^*^ | .00055 | .000 | -.0079 | -.0055 |  |
|  | Effect of Molasses against CDW (g/L) for *Enterobacter* sp. DSAAI-4 growth | -.00741^*^ | .00055 | .000 | -.0086 | -.0062 |  |
|  | Effect of Barley bran against CDW (g/L) for *Enterobacter* sp. DSAAI-4 growth | -.00891^*^ | .00055 | .000 | -.0101 | -.0077 |  |
|  | Effect of Wheat bran against CDW (g/L) for Enterobacter sp. DSAAI-4 growth | -.00068 | .00055 | .236 | -.0019 | .0005 |  |
|  | Effect of Activated sludge against CDW (g/L) for *Enterobacter* sp. DSAAI-4 growth  *Enterobacter* sp. DSAAI-4 growth | -.00799^*^ | .00055 | .000 | -.0092 | -.0068 |  |
|  |  |  |  |  |  |  |  |
| Effect of Activated sludge against CDW (g/L) for *Enterobacter* sp. DSAAI-4 growth | Effect of Teff straw against CDW (g/L) for *Enterobacter* sp. DSAAI-4 growth | .00700^*^ | .00055 | .000 | .0058 | .0082 |  |
|  | Effect of Bagasse against CDW (g/L) for *Enterobacter* sp. DSAAI-4 growth | .00128^*^ | .00055 | .036 | .0001 | .0025 |  |
|  | Effect of Molasses against CDW (g/L) for *Enterobacter* sp. DSAAI-4 growth | .00058 | .00055 | .313 | -.0006 | .0018 |  |
|  | Effect of Barley bran against CDW (g/L) for *Enterobacter* sp. DSAAI-4 growth | -.00092 | .00055 | .016 | -.0021 | .0003 |  |
|  | Effect of Wheat bran against CDW (g/L) for Enterobacter sp. DSAAI-4 growth | .00730^*^ | .00055 | .000 | .0061 | .0085 |  |
|  | Effect of Cotton waste against CDW (g/L) for Enterobacter sp. DSAAI-4 growth | .00799^*^ | .00055 | .000 | .0068 | .0092 |  |
| *. The mean difference is significant at the 0.05 level. | |  |  |  |  |  |  |
